# Supplementary material for: A Staphylococcus pro-apoptotic peptide induces acute exacerbation of pulmonary fibrosis
Source: Nat Commun. 2020 Mar 24;11:1539. doi: 10.1038/s41467-020-15344-3 (PMC7093394; doi:10.1038/s41467-020-15344-3)
Supplement: Supplementary file 1 — Supplementary Information [file 41467_2020_15344_MOESM1_ESM.pdf]

Supplementary Information

A Staphylococcus pro-apoptotic peptide induces acute exacerbation of pulmonary fibrosis

D'Alessandro-Gabazza *et al*.

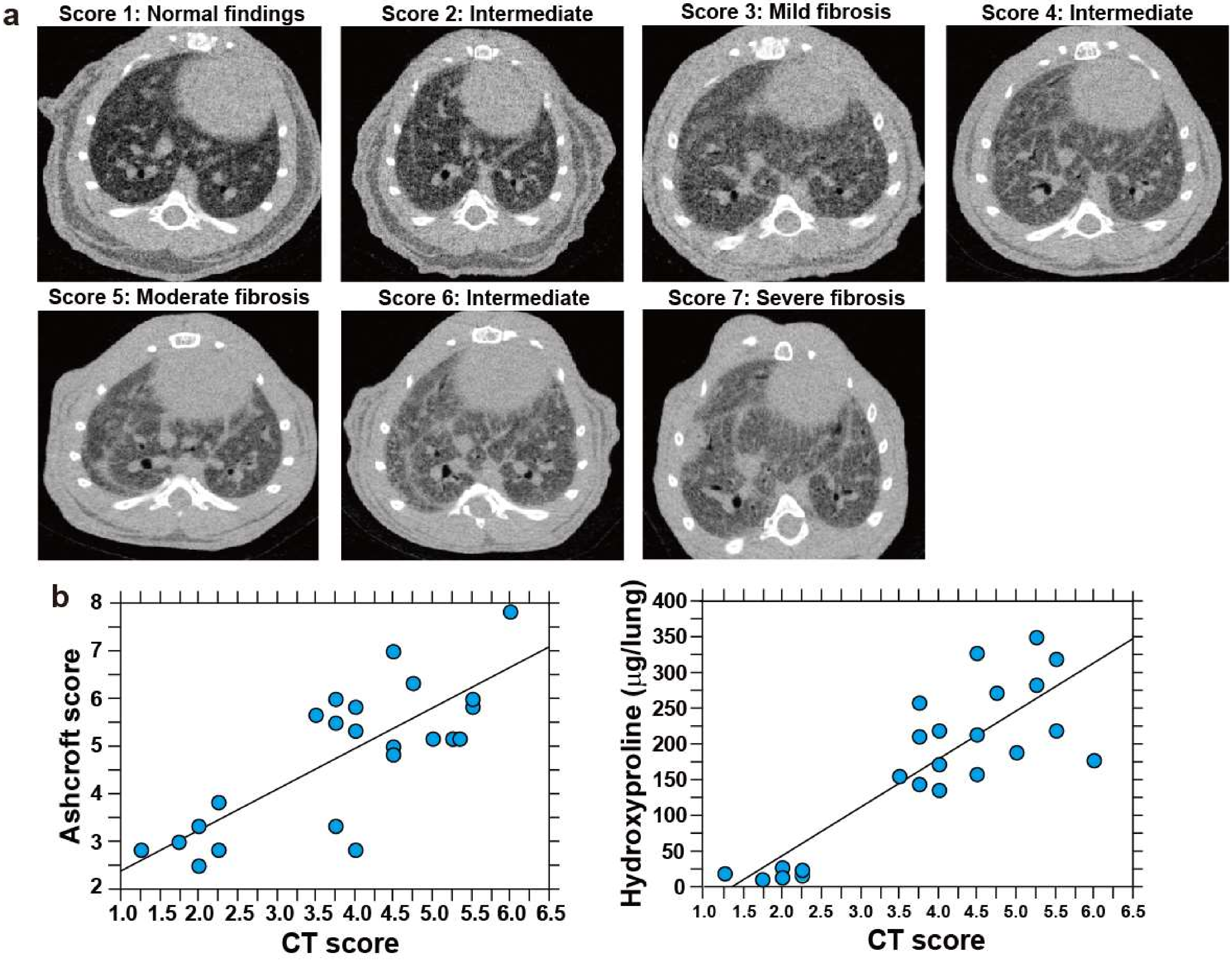

a
Score 1: Normal findings
Score 2: Intermediate
Score 3: Mild fibrosis
Score 4: Intermediate
Score 5: Moderate fibrosis
Score 6: Intermediate
Score 7: Severe fibrosis
b
Ashcroft score
CT score
Hydroxyproline (μg/lung)
CT score

Supplementary Figure 1. Criteria for scoring lung radiological findings and correlation of CT score with the Ashcroft fibrosis score and with the hydroxyproline content of the lungs. a, Computed tomography (CT) was performed as described under methods. Criteria for scoring CT findings were as follows: score 1: normal findings; score 2, intermediate; score 3; mild fibrosis; score 4: intermediate; score 5, moderate fibrosis; score 6: intermediate; and score 7, severe fibrosis. The average of scores of six pulmonologists was taken as the CT score of an individual mouse. **b**, The Ashcroft fibrosis score and the hydroxyproline content were measured as described under methods. Male mice with 20 to 25 g of body weight and 10-week of age were used in the experiments. N=23 mice. The CT score was significantly correlated with the Ashcroft score ($r=0.78$; $p<0.0001$) and with the hydroxyproline content of the lungs ($r=0.84$; $p<0.0001$). Statistical analysis by Pearson-product moment correlation. The source data underlying Supplementary Fig 1b are provided in the Source Data file.

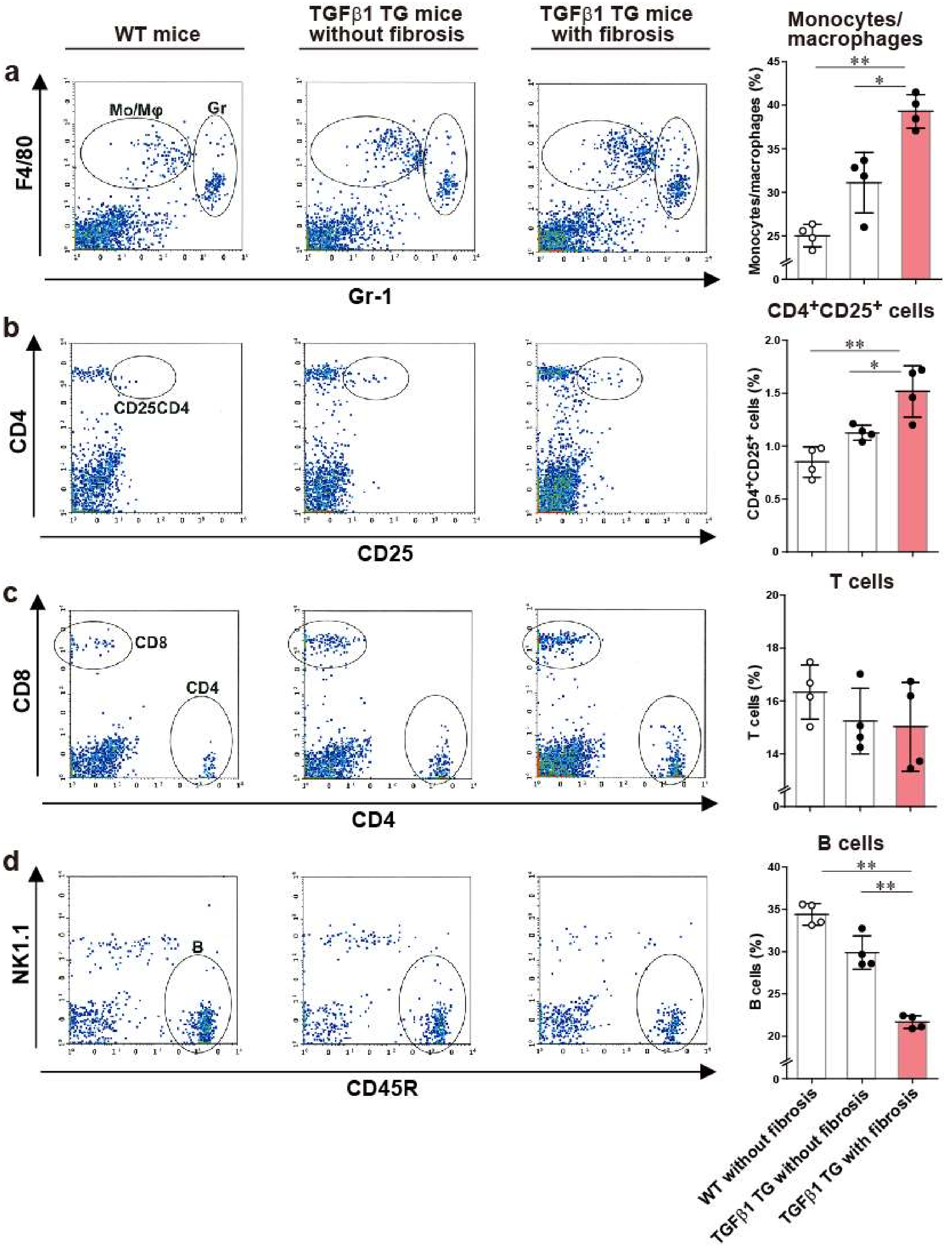

Supplementary Figure 2. Abnormal immune response in lung fibrotic tissue. The percentage of monocytes/macrophages (**a**), CD4Cd25 cells (**b**), T cells (**c**) and B cells (**d**) in lung fibrotic tissue from wild-type (WT) mice (n=4), transforming growth factor (TGF)β1 transgenic (TG) mice with (n=4) and without (n=4) fibrosis was counted by flow cytometry using specific antibodies as described under methods. Bars indicate the means ± S.D. Statistical analysis by ANOVA with Tukey's test. *p<0.05, **p<0.01. The source data underlying Supplementary Figs 2a-d are provided in the Source Data file.

Supplementary Table 1. Immune cells in lung tissue of each group of mouse

Immune cells (%)	WT mice	TGFβ1 TG mice without fibrosis	TGFβ1 TG mice with fibrosis
Monocytes/Macrophages	25.00 ± 1.28	31.09 ± 3.48*	39.30 ± 1.93**†
Granulocytes	11.59 ± 1.18	11.26 ± 0.89	12.54 ± 1.10
Dendritic cells	7.00 ± 0.30	6.86 ± 0.82	7.17 ± 0.70
Total lymphocytes	56.41 ± 1.30	50.80 ± 2.73*	41.00 ± 1.94**‡
B cells	34.41 ±1.29	29.87 ± 1.98*	21.67 ± 0.76**‡
T cells	16.33 ± 1.03	15.24 ± 1.23	15.02 ± 1.68
Natural killer cells	5.27 ± 0.51	4.80 ± 0.31	2.85 ± 0.43**‡
Natural killer T cells	0.40 ± 0.11	0.88 ± 0.42	1.45 ± 0.34*
$CD4^+$ T cells	9.44 ± 0.18	9.11 ± 1.42	9.26 ± 0.84
$CD8^+$ T cells	6.75 ± 0.99	6.12 ± 0.50	6.39 ± 0.53
$CD4^+CD25^+$	0.85 ± 0.14	1.12 ± 0.07	1.52 ± 0.24**†
γ/δ T cells	0.53 ± 0.11	0.52 ± 0.11	0.74 ± 0.10*†
B/T cells ratio	2.12 ± 0.19	1.96 ± 0.03	1.46 ± 0.16**†
CD4/CD8 ratio	1.43 ± 0.25	1.50 ± 0.32	1.45 ± 0.05

Data are the means ± S.D. Number of cells are expressed as the percentage of total number of lung cells. Each mouse group with n=4. Statistical analysis by ANOVA with Tukey's test *p<0.05 or **p<0.01 vs WT mice; †p<0.05 or ‡p<0.05 vs TGFβ1 TG mice without fibrosis. TGFβ1, transforming growth factorβ1. WT, wild type. Source data are provided in the Source Data file.

Supplementary Table 2. Expression of fibrotic factors and sodium channels in lung tissues from wild-type and transgenic mice with and without lung fibrosis

Variables	WT	TGFβ1 TG without fibrosis	TGFβ1 TG with fibrosis
mRNA relative level			
Ctfr	0.965 ± 0.057	0.720 ± 0.118	0.492 ± 0.135*†
Scnn1γ	0.910 ± 0.117	0.817 ± 0.117	0.495 ± 0.135*†
Scnn1β	1.198 ± 0.212	0.971 ± 0.276	0.612 ± 0.094*
Scnn1α	0.100 ± 0.317	0.995 ± 0.167	0.845 ± 0.218*
TNFα	0.486 ± 0.046	0.486 ± 0.102	0.893 ± 0.084*†
IFNγ	0.745 ± 0.161	0.540 ± 0.078	1.162 ± 0.187*†
Periostin	0.860 ± 0.1396	0.1396 ± 0.911	1.099 ± 0.027
Ctgf	0.822 ± 0.103	0.734 ± 0.039	1.186 ± 0.026*†
mTGFβ1	0.558 ± 0.046	0.520± 0.054	0.792 ± 0.067*†
Vegf	0.630 0.114	0.542 0.181	1.020 0.263*†
INOS	0.718 0.159	0.755 0.097	1.235 0.057*†
Mcp-1	0.695 ± 0.154	0.754 ± 0.109	1.040 ± 0.065*†
αSMA	0.740 ± 0.078	0.666 ± 0.093	0.837 ± 0.140
Fn1	0.801 ± 0.096	0.678 ± 0.092	1.097 ± 0.129*†
Col1α1	0.759 ± 0.074	0.493 ± 0.080	1.069 ± 0.220*†
Plasma active TGFβ1	97.542 ± 19.136	246.165 ± 94.132	365.897 ± 58.751*
Plasma total TGFβ1	1521.586 ± 645.522	3858.940 ± 1973.896	8086.258 ± 838.130*†

Data are expressed as the means ± S.D. Each mouse group with n=4. Statistical analysis by ANOVA with Tukey's test. *p<0.05 vs WT; †p<0.05 vs TGFβ1 TG mouse without fibrosis. Ctfr, cystic fibrosis transmembrane conductance regulator; Scnn1γ, sodium channel epithelial 1 γ subunit; Scnn1β, sodium channel epithelial 1 β subunit; Scnn1α, sodium channel epithelial 1 α subunit; TNFα, tumor necrosis factorα; IFNγ, interferonγ; Ctgf, connective tissue growth factor; mTGFβ1, mouse transforming growth factor β1; Vegf, vascular epithelial growth factor; iNOS, inducible nitric oxide synthase; Mcp-1, monocyte chemoattractant protein-1; αSMA, αsmooth muscle actin; Fn1, fibronectin 1; Col1α1, collagen 1α1. WT, wild-type; TG, transgenic. Source data are provided in the Source Data file.

	Lung tissue Na$^+$	Plasma Total Tgfβ1	Plasma active Tgfβ1	Col1a1 mRNA	Fn1 mRNA	α-SMA mRNA	Periostin mRNA	Ctgf mRNA	Mcp-1 mRNA	Ifn-γ mRNA	Tnf-α mRNA	mTgfβ1 mRNA	Vegf mRNA	iNOS mRNA	Scnn1α mRNA	Scnn1β mRNA	Scnn1γ mRNA	Ctfr mRNA	Monocytes/ macrophages	Total lymphocytes	B lymphocytes	%CD25$^+$CD$^+$ T cells
Lung tissue Na$^+$	1.0																					
Plasma Total Tgfβ1	0.7*	1.0																				
Plasma active Tgfβ1	0.7*	0.9*	1.0																			
Col1a1 mRNA	0.6*	0.4	0.3	1.0																		
Fn1 mRNA	0.6*	0.5	0.4	0.9*	1.0																	
α-SMA mRNA	0.5	0.2	0.3	0.8*	0.6*	1.0																
Periostin mRNA	0.7*	0.6*	0.5	0.5	0.6*	0.5	1.0															
Ctgf mRNA	0.7*	0.6*	0.5	0.8*	0.7*	0.4	0.6*	1.0														
Mcp-1 mRNA	0.6*	0.6*	0.6*	0.5	0.5	0.3	0.4	0.5	1.0													
Ifn-γ mRNA	0.7*	0.4	0.3	0.9*	0.9*	0.7*	0.6*	0.8*	0.4	1.0												
Tnf-α mRNA	0.5	0.6*	0.5	0.6*	0.6*	0.3	0.5	0.7*	0.8*	0.6*	1.0											
mTgfβ1 mRNA	0.7*	0.7*	0.6*	0.8*	0.9*	0.6*	0.5	0.7*	0.7*	0.8*	0.7*	1.0										
Vegf mRNA	0.3	0.4	0.4	0.3	0.3	0.1	0.1	0.3	0.6*	0.4	0.7*	0.6*	1.0									
iNOS mRNA	0.4	-0.7*	-0.7*	0.5	0.5	0.4	0.3	0.5	-0.9*	0.5	0.9*	0.7*	0.7*	1.0								
Scnn1α mRNA	-0.5	-0.6*	-0.5	-0.2	-0.3	-0.1	-0.1	-0.3	-0.3	-0.2	-0.3	-0.2	-0.1	-0.4	1.0							
Scnn1β mRNA	-0.6*	-0.6*	-0.4	-0.4	-0.4	-0.1	-0.4	-0.5	-0.6*	-0.5	-0.5	-0.3	-0.1	-0.5	0.7*	1.0						
Scnn1γ mRNA	-0.8*	-0.7*	-0.6*	-0.5	-0.5	-0.3	-0.5	-0.5	-0.7*	-0.5	-0.5	-0.5	-0.3	-0.5	0.8*	0.8*	1.0					
Ctfr mRNA	-0.6*	-0.7*	-0.7*	-0.3	-0.3	-0.1	-0.4	-0.4	-0.7*	-0.3	-0.5	-0.3	-0.3	-0.7*	0.7*	0.8*	0.7*	1.0				
Monocytes/ macrophages	0.7*	0.9*	0.8*	0.4	0.4	0.4	0.5	0.5	0.8*	0.4	0.7*	0.6*	0.6*	0.9*	-0.6*	-0.6*	-0.7*	-0.8*	1.0			
Total lymphocytes	-0.7*	-0.8*	-0.7*	-0.4	-0.5	-0.4	-0.6*	-0.4	-0.8*	-0.4	-0.7*	-0.7*	-0.5	-0.8*	0.6*	0.6*	0.7*	0.8*	-0.9*	1.0		
B lymphocytes	-0.7*	-0.8*	-0.8*	-0.4	-0.5	-0.4	-0.6*	-0.5	-0.7*	-0.4	-0.6*	-0.6*	-0.5	-0.8*	0.6*	0.6*	0.7*	0.8*	-0.9*	0.9*	1.0	
CD4$^+$CD25$^+$ T cells	0.6*	0.8*	0.7*	0.3	0.4	0.2	0.6*	0.5	0.8*	0.3	0.7*	0.5	0.3	0.7*	-0.6*	-0.7*	-0.7*	-0.8*	0.8*	-0.9*	-0.9*	1.0

Supplementary Figure 3. The level of sodium correlates with the number of immune cells, and with the expression of fibrotic markers and sodium channels in lung fibrotic tissues. The concentration of sodium, the expression of fibrotic factors, pro-fibrotic cytokines, chemokines, angiogenic factors and the percentage of immune cells in lung tissue were assessed in lung tissue from wild-type (n=4) and transforming growth factor (TGF)β1 transgenic (TG) mice with (n=4) and without (n=4) lung fibrosis. Spearman correlation r values are shown. Ctfr, cystic fibrosis transmembrane conductance regulator; Scnn1α, sodium channel epithelial 1 α subunit; Scnn1β, sodium channel epithelial 1 β subunit; Scnn1γ, sodium channel epithelial 1 γ subunit; TNFα, tumor necrosis factorα; IFNγ, interferonγ; Ctgf, connective tissue growth factor; mTGFβ1, mouse transforming growth factor β1; Vegf, vascular epithelial growth factor; iNOS, inducible nitric oxide synthase; Mcp-1, monocyte chemoattractant protein-1; αSMA, αsmooth muscle actin; Fn1, fibronectin 1; Col1α1, collagen 1α1. Statistical analysis by Spearman correlation. $*p<0.05$. Source data are provided in the Source Data file.

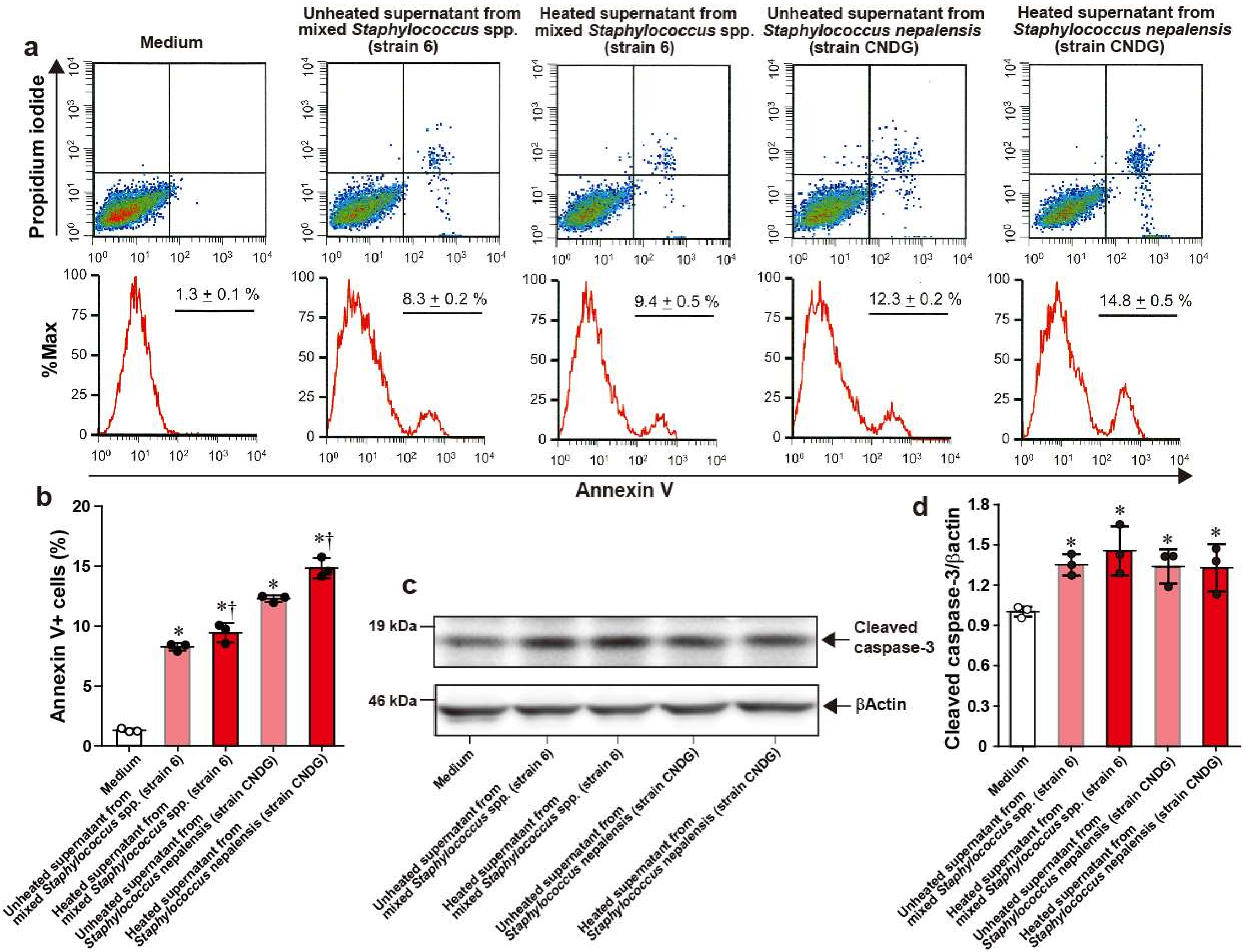

a
Medium
Unheated supernatant from mixed Staphylococcus spp. (strain 6)
Heated supernatant from mixed Staphylococcus spp. (strain 6)
Unheated supernatant from Staphylococcus nepalensis (strain CNDG)
Heated supernatant from Staphylococcus nepalensis (strain CNDG)
Propidium iodide
%Max
1.3 ± 0.1 %
8.3 ± 0.2 %
9.4 ± 0.5 %
12.3 ± 0.2 %
14.8 ± 0.5 %
Annexin V
b
Annexin V+ cells (%)
c
19 kDa
46 kDa
Cleaved caspase-3
βActin
d
Cleaved caspase-3/βactin
Medium
Unheated supernatant from mixed Staphylococcus spp. (strain 6)
Heated supernatant from mixed Staphylococcus spp. (strain 6)
Unheated supernatant from Staphylococcus nepalensis (strain CNDG)
Heated supernatant from Staphylococcus nepalensis (strain CNDG)

Supplementary Figure 4. The pro-apoptotic factor in culture supernatant from bacteria is heat-stable. Culture supernatant from bacteria was incubated at 85ºC for 15 min before adding to the culture medium of A549 alveolar epithelial cells at 1/10 dilution.. **a,b,** Flow cytometry of A549 cells was performed after staining with propidium iodide and annexin V. Each group with n=3. Bars indicate the means ± S.D. Statistical analysis by ANOVA with Newman-Keuls test. *p<0.001, vs medium; †p<0.05 vs unheated supernatant from *Staphylococcus nepalensis* (strain CNDG) or from strain 6. **c**,**d**, Activation of caspase-3 by the culture supernatant as evaluated by Western blotting after stimulating A549 alveolar epithelial cells in the presence of medium or supernatant of the mixture of *Staphylococcus* spp. or *Staphylococcus nepalensis* strain CNDG. Each group with n=3. Bars indicate the means ± S.D. Statistical analysis by ANOVA with Newman-Keuls test. *p<0.05 vs medium. The source data underlying Supplementary Figs 4b,c,d are provided in the Source Data file.

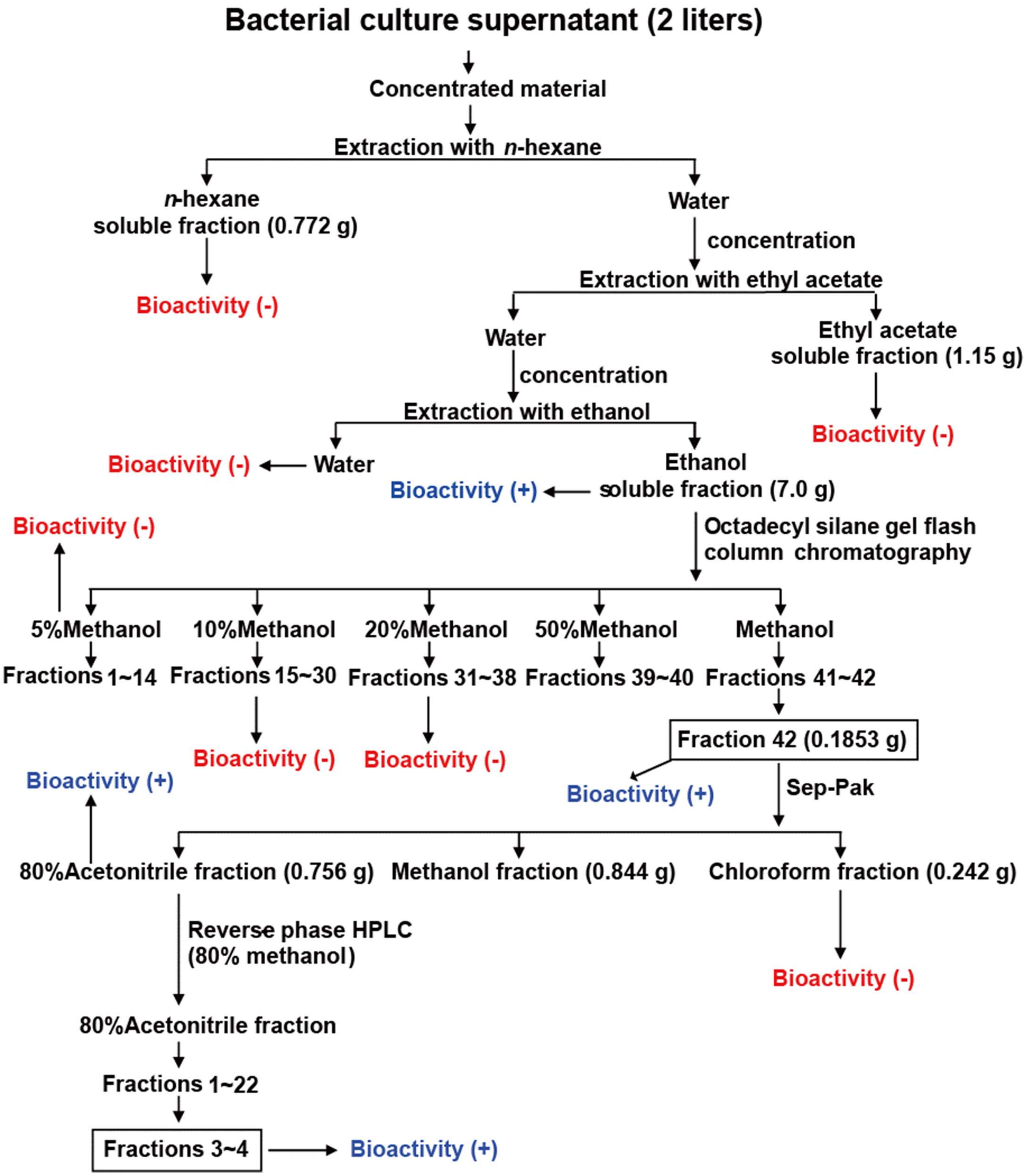

Supplementary Figure 5. Schematic diagram describing the sample fractionation steps and the bioactivity of each fraction. Fractionation of the culture supernatant was performed as described under methods. The pro-apoptotic activity of the fraction on A549 alveolar epithelial cells was evaluated by flow cytometry and it is indicated as bioactivity (+) or no bioactivity (-). Source data are provided in the Source Data file.

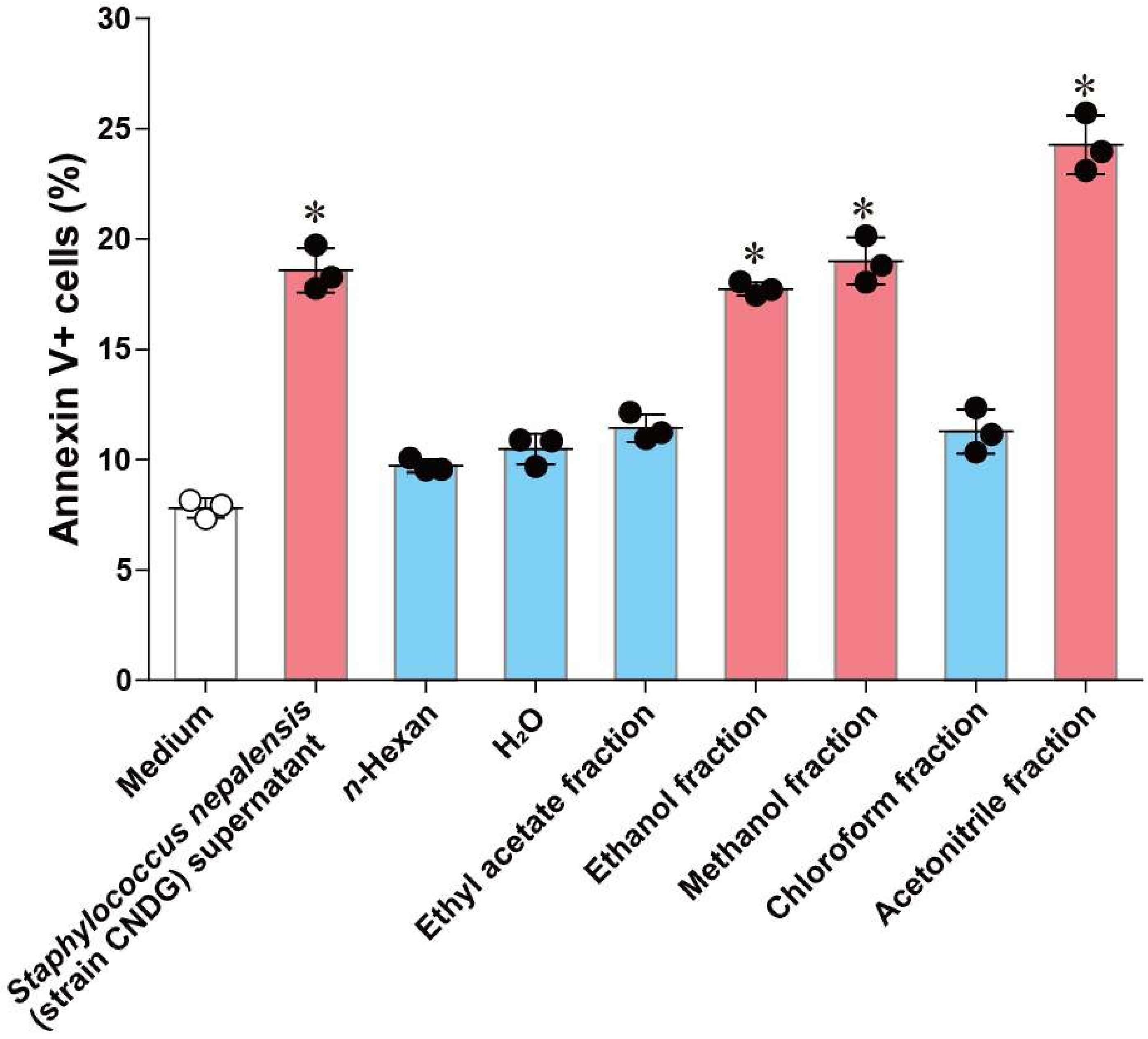

Supplementary Figure 6. Pro-apoptotic activity of each fraction, obtained during fractionation of bacterial supernatant from *Staphylococcus nepalensis*, on A549 alveolar epithelial cells. Fractionation of the culture supernatant was performed as described under methods. The pro-apoptotic activity of the fraction was evaluated by flow cytometry. Each group with n=3. Bars indicate the means ± S.D. Statistical analysis by ANOVA with Tukey's test. *p<0.001 vs medium. Source data are provided in the Source Data file.

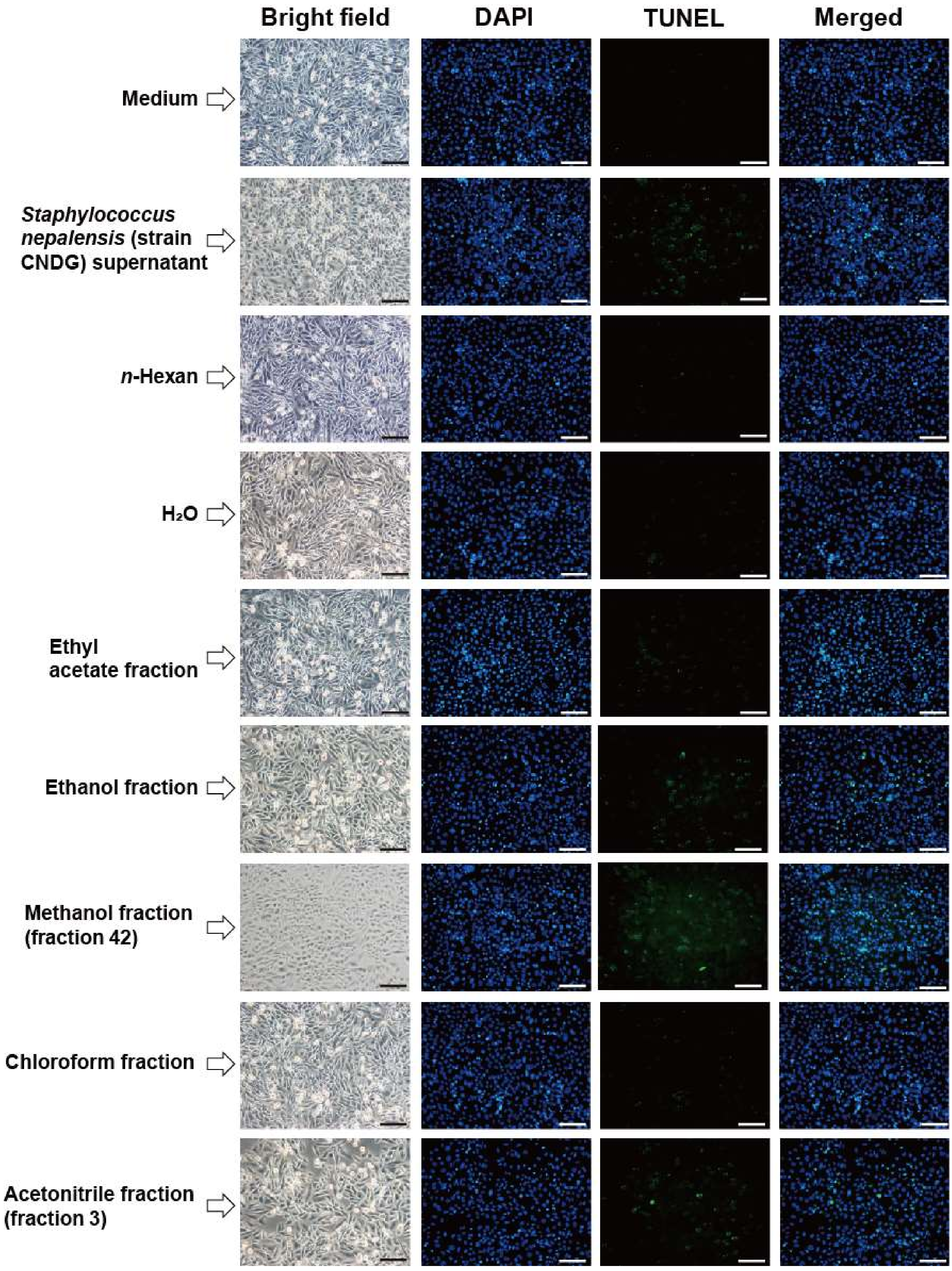
Bright field
DAPI
TUNEL
Merged
Medium
Staphylococcus nepalensis (strain CNDG) supernatant
n-Hexan
H_2O
Ethyl acetate fraction
Ethanol fraction
Methanol fraction (fraction 42)
Chloroform fraction
Acetonitrile fraction (fraction 3)

Supplementary Figure 7. Ethanol, methanol and acetonitrile fractions of the culture supernatants of *Staphylococcus nepalensis* strain CNDG induces apoptosis of lung epithelial cells. A549 lung epithelial cells were cultured in the presence of each fraction for 48h and apoptosis was evaluated by terminal deoxynucleotidyl transferase dUTP nick end labeling (TUNEL) assay. DAPI, 4',6-diamidino-2-phenylindole. Representative microphotographs out of two experiments are shown. Scale bars indicate 100 μm.

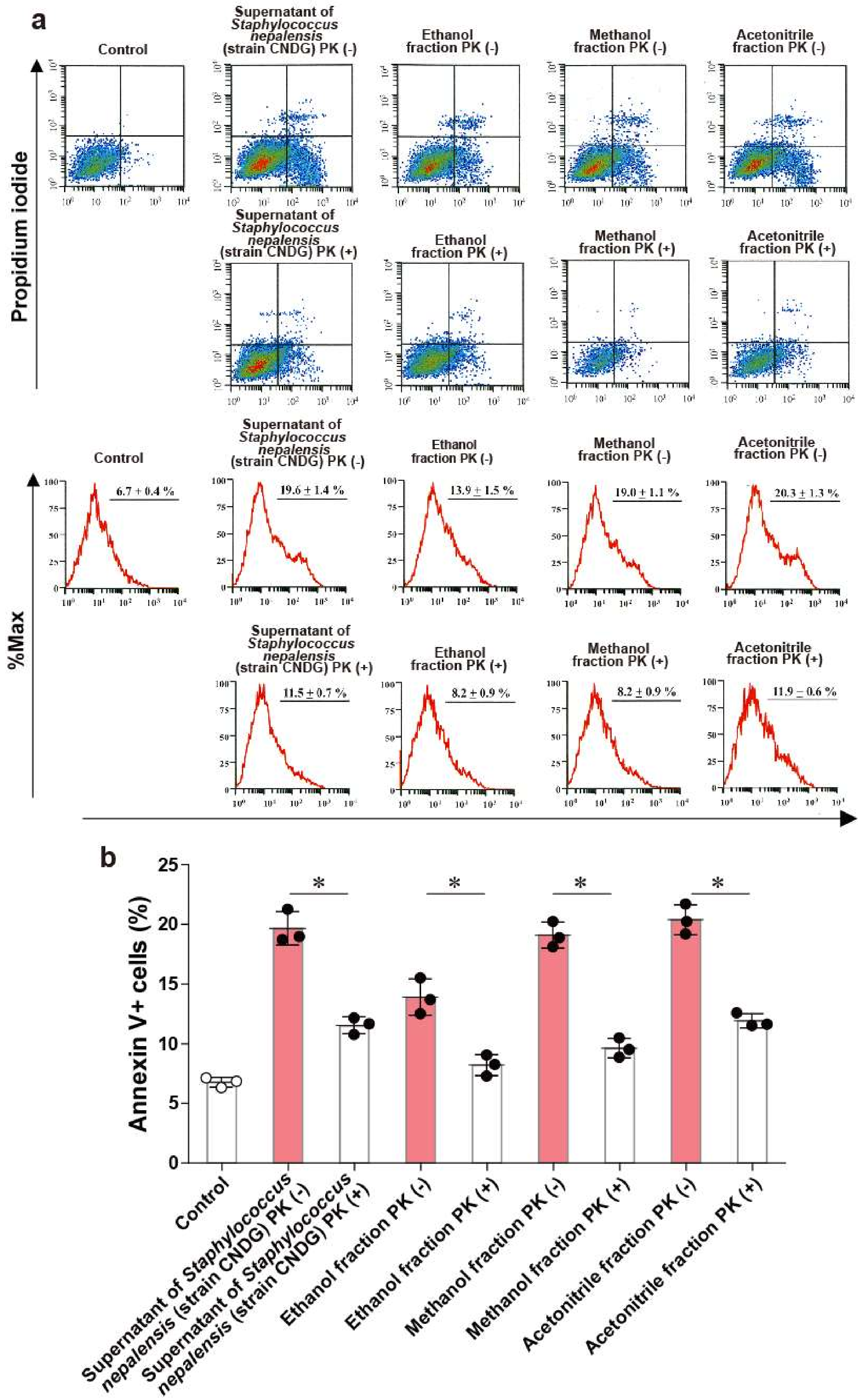

a
Control
Supernatant of Staphylococcus nepalensis (strain CNDG) PK (-)
Ethanol fraction PK (-)
Methanol fraction PK (-)
Acetonitrile fraction PK (-)
Supernatant of Staphylococcus nepalensis (strain CNDG) PK (+)
Ethanol fraction PK (+)
Methanol fraction PK (+)
Acetonitrile fraction PK (+)
Propidium iodide
%Max
6.7 ± 0.4 %
19.6 ± 1.4 %
13.9 ± 1.5 %
19.0 ± 1.1 %
20.3 ± 1.3 %
11.5 ± 0.7 %
8.2 ± 0.9 %
8.2 ± 0.9 %
11.9 ± 0.6 %
b
Annexin V+ cells (%)
Control
Supernatant of Staphylococcus nepalensis (strain CNDG) PK (-)
Supernatant of Staphylococcus nepalensis (strain CNDG) PK (+)
Ethanol fraction PK (-)
Ethanol fraction PK (+)
Methanol fraction PK (-)
Methanol fraction PK (+)
Acetonitrile fraction PK (-)
Acetonitrile fraction PK (+)

Supplementary Figure 8. The pro-apoptotic activity of the fractions obtained from the supernatants of cultured *Staphylococcus nepalensis* strain CNDG is sensitive to proteinase K treatment. Culture supernatant and ethanol, methanol or acetonitrile fractions of the culture supernatant from *Staphylococcus nepalensis* were incubated in the presence of 200 μg/ml of proteinase K (PK) at 37 ºC before adding to the culture medium of A549 alveolar epithelial cells at 1/10 dilution. Each group with n=3. **a**,**b**, Flow cytometry of A549 alveolar epithelial cells was performed after staining with propidium iodide and annexin V. Bars indicate the means ± S.D. Statistical analysis by ANOVA with Tukey's test. *p<0.01. Abbreviations: PK, proteinase K. The source data underlying Supplementary Fig 8b are provided in the Source Data file.

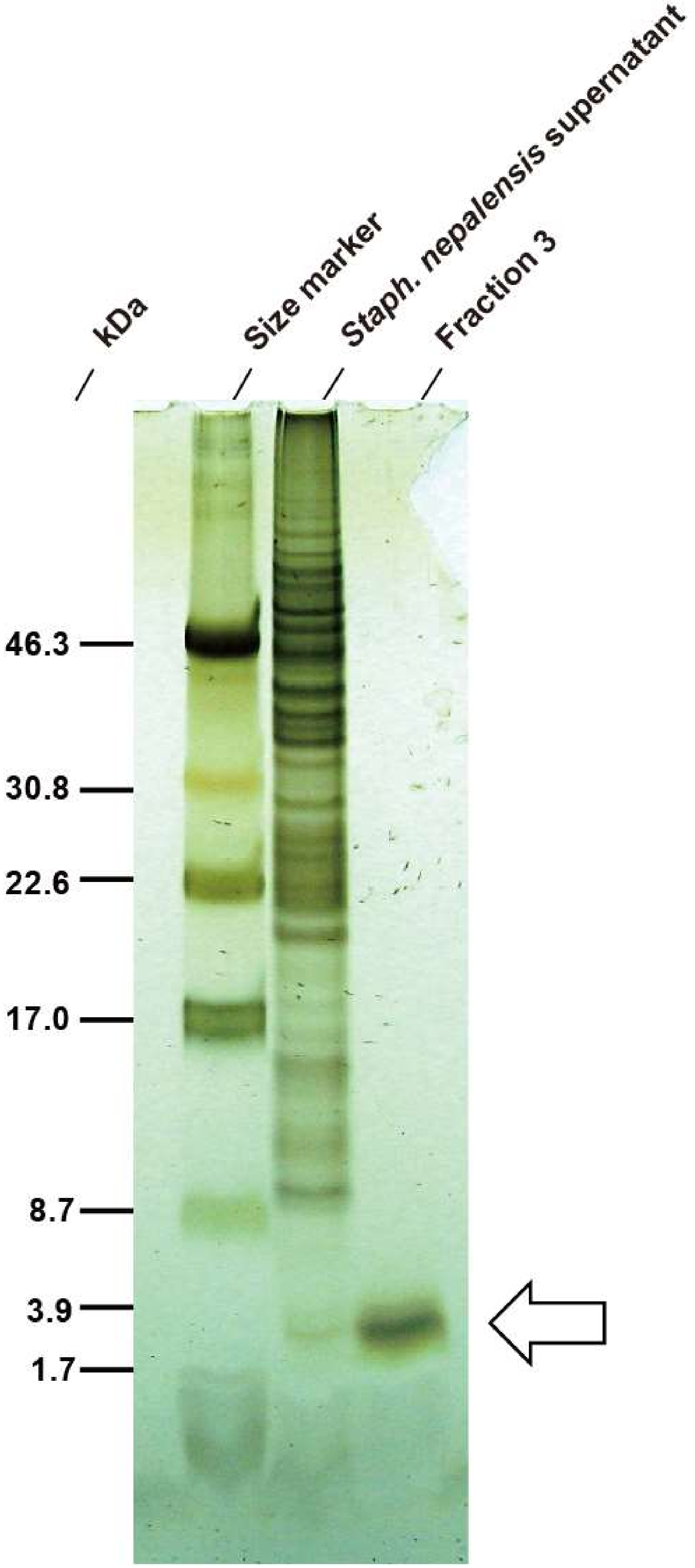

Supplementary Figure 9. Silver staining of the fraction showing pro-apoptotic activity. Five micrograms of the high performance liquid chromatography fraction (fraction 3) with biological activity was loaded on a 15% sodium dodecyl sulfate polyacrylamide gel and silver-staining was performed using a commercial kit. Representative microphotographs out of three experiments with similar results are shown.

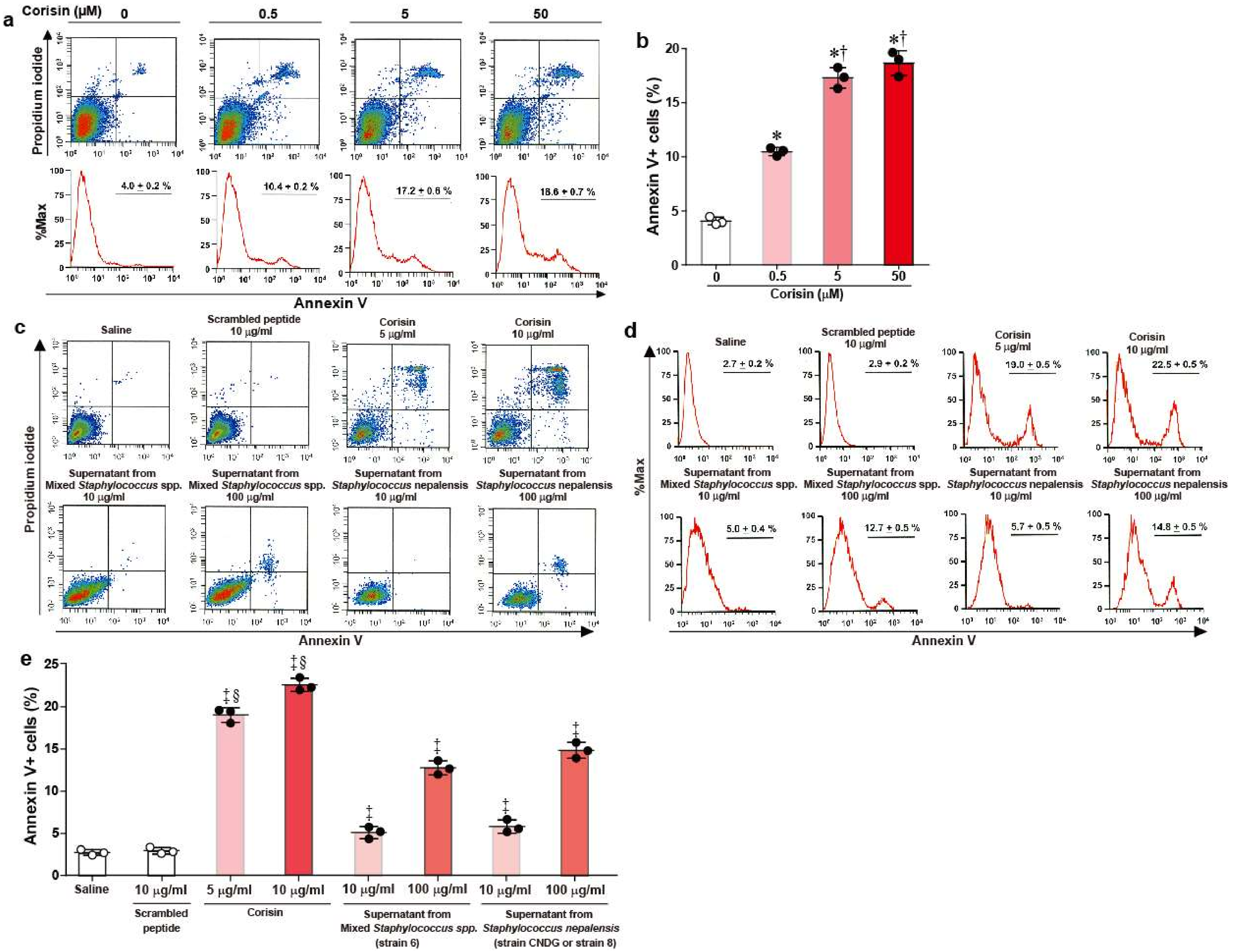
a
Corisin (μM)
0
0.5
5
50
Propidium iodide
%Max
4.0 ± 0.2 %
10.4 ± 0.2 %
17.2 ± 0.6 %
18.6 ± 0.7 %
Annexin V
b
Annexin V+ cells (%)
Corisin (μM)
c
Saline
Scrambled peptide 10 μg/ml
Corisin 5 μg/ml
Corisin 10 μg/ml
Supernatant from Mixed Staphylococcus spp. 10 μg/ml
Supernatant from Mixed Staphylococcus spp. 100 μg/ml
Supernatant from Staphylococcus nepalensis 10 μg/ml
Supernatant from Staphylococcus nepalensis 100 μg/ml
Propidium iodide
Annexin V
d
%Max
2.7 ± 0.2 %
2.9 ± 0.2 %
19.0 ± 0.5 %
22.5 ± 0.5 %
5.0 ± 0.4 %
12.7 ± 0.5 %
5.7 ± 0.5 %
14.8 ± 0.5 %
Annexin V
e
Annexin V+ cells (%)
Saline
10 μg/ml
5 μg/ml
10 μg/ml
10 μg/ml
100 μg/ml
10 μg/ml
100 μg/ml
Scrambled peptide
Corisin
Supernatant from Mixed Staphylococcus spp. (strain 6)
Supernatant from Staphylococcus nepalensis (strain CNDG or strain 8)

Supplementary Figure 10. Synthetic peptide prepared by a different manufacturer (ThermoFisher Scientific, Waltham, MA) induces dose-dependent apoptosis of alveolar epithelial cells, and the apoptotic activity of corisin was significantly more potent than equal concentration of supernatant protein. **a**,**b**, Flow cytometry analysis of A549 alveolar epithelial cells cultured for 48h in DMEM medium containing varying concentration of corisin. Each group with n=3. Bars indicate the means ± S.D. Statistical analysis by ANOVA with Tukey's test. *p<0.001 vs control (0 μg/ml); †p<0.001 vs 0.5 μg/ml of corisin. **c**,**d**,**e**, Flow cytometry analysis of A549 alveolar epithelial cells cultured for 48h in DMEM medium containing varying concentrations of corisin (5 or 10 μg/ml), supernatant protein from mixed *Staphylococcus* spp. or strain 6 (10 or 100 μg/ml), or from *Staphylococcus nepalensis* strain CNDG or strain 8 (10 or 100 μg/ml). Each group with n=3. Bars indicate the means ± S.D. Statistical analysis by ANOVA and Tukey's test. ‡p<0.05 vs saline or scrambled peptide; §p<0.001 vs supernatant protein (10 and 100 μg/ml) from mixed *Staphylococcus* spp. or *Staphylococcus nepalensis*. The source data underlying Supplementary Figs 10b,e are provided in the Source Data file.

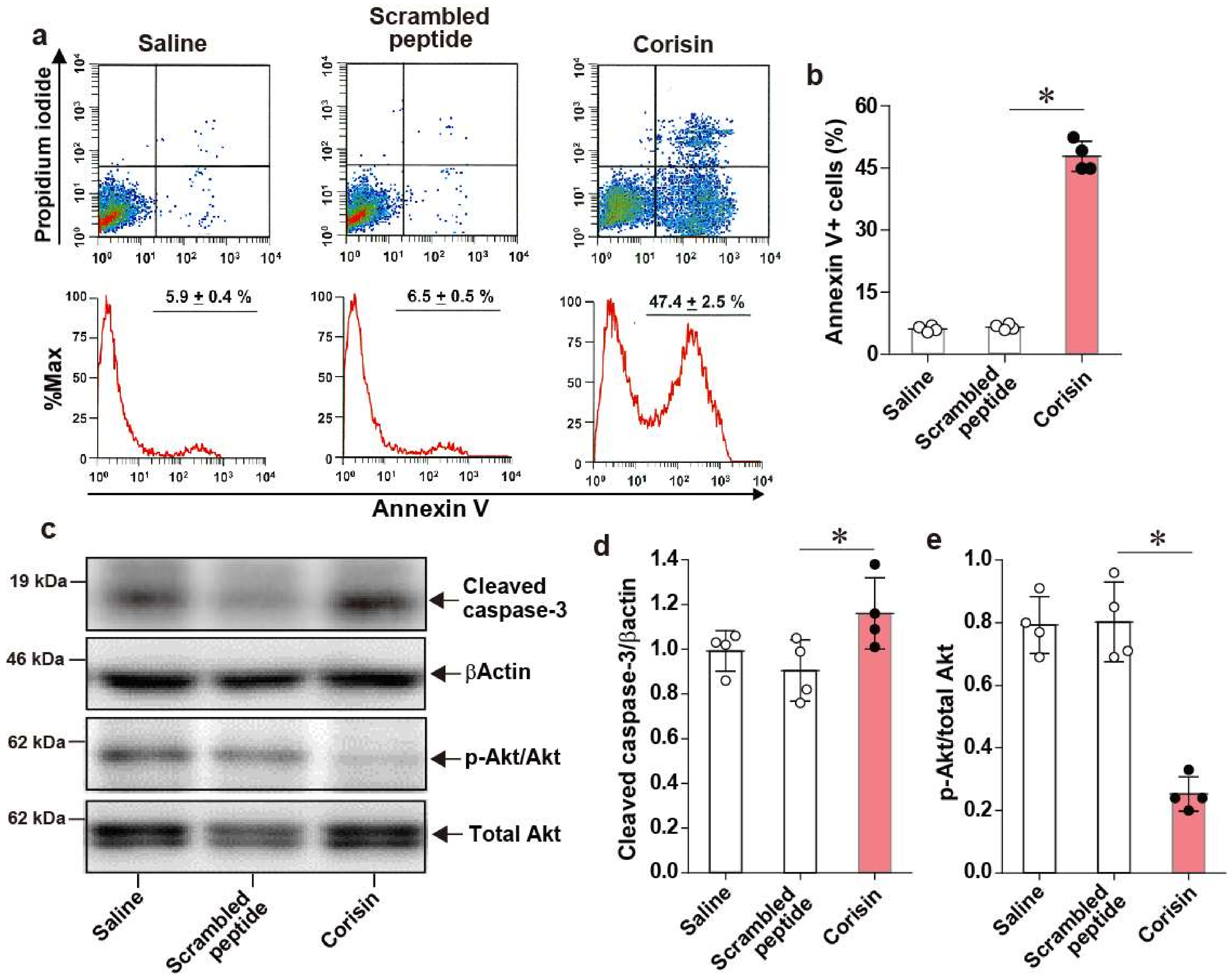

a
Saline
Scrambled peptide
Corisin
Propidium iodide
%Max
5.9 ± 0.4 %
6.5 ± 0.5 %
47.4 ± 2.5 %
Annexin V
b
Annexin V+ cells (%)
Saline
Scrambled peptide
Corisin
c
19 kDa
46 kDa
62 kDa
62 kDa
Cleaved caspase-3
βActin
p-Akt/Akt
Total Akt
Saline
Scrambled peptide
Corisin
d
Cleaved caspase-3/βactin
e
p-Akt/total Akt

Supplementary Figure 11. The pro-apoptotic peptide (corisin) but not its scrambled sequence induces apoptosis of normal human bronchial epithelial cells. a,b, Flow cytometry analysis of normal human bronchial epithelial (NHBE) cells cultured for 48h in DMEM medium containing 10 μM of corisin or of its scrambled sequence. Each treatment group with n=4. Bars indicate the means ± S.D. Statistical analysis by ANOVA with Tukey's test. *p<0.001. **c**, Western blotting of lysates of NHBE cells treated with corisin or scrambled peptide. Each treatment group with n=4. Representative blot of each treatment group is shown. **d**,**e**, The intensity of the Western blot membrane bands was quantified by densitometry using the ImageJ software. Each treatment group with n=4. Bars indicate the means ± S.D. Statistical analysis by one-tailed Mann-Whitney U test. *p<0.05. The source data underlying Supplementary Figs 11b,d,e are provided in the Source Data file.

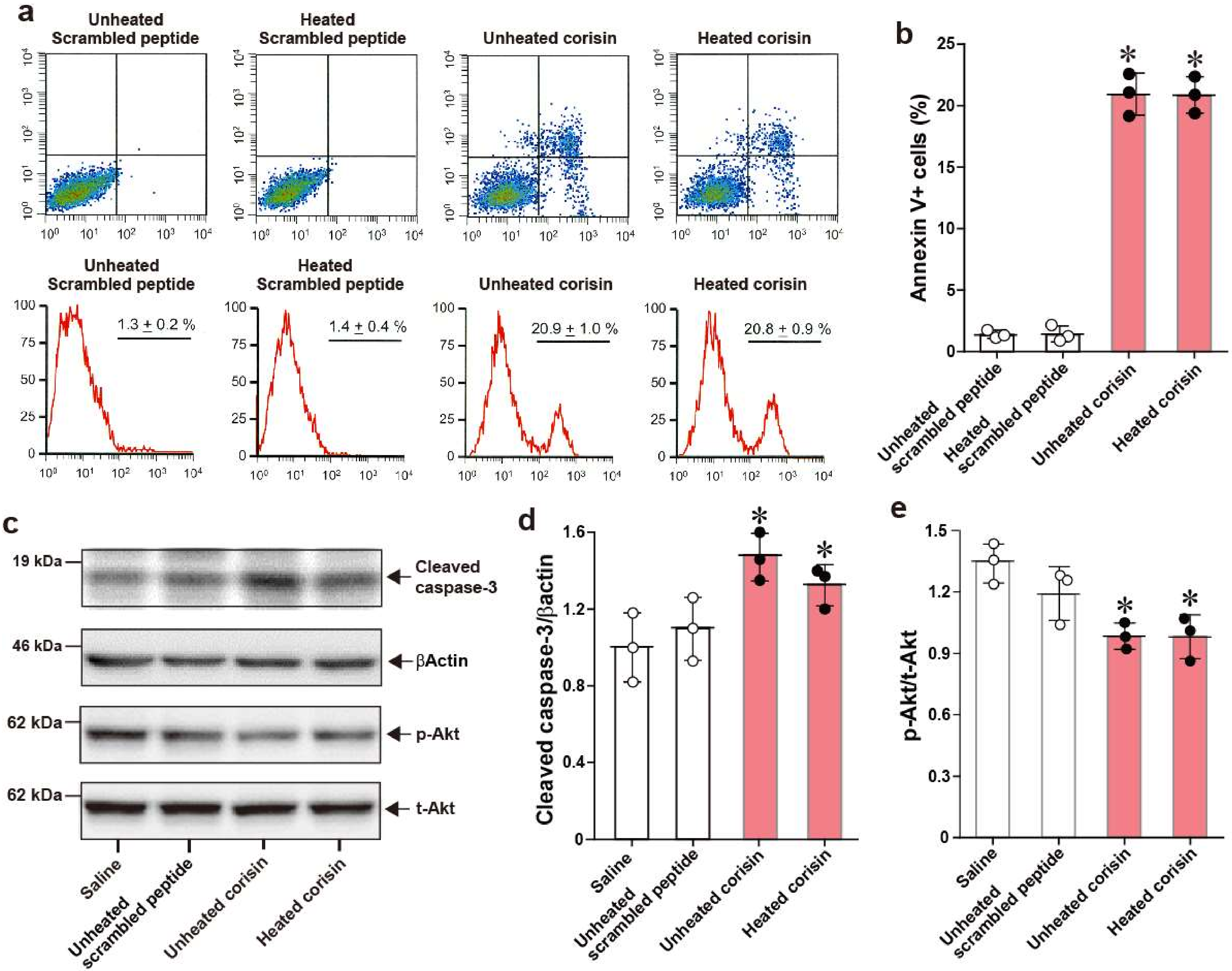
a
Unheated Scrambled peptide
Heated Scrambled peptide
Unheated corisin
Heated corisin
1.3 ± 0.2 %
1.4 ± 0.4 %
20.9 ± 1.0 %
20.8 ± 0.9 %
b
Annexin V+ cells (%)
Unheated scrambled peptide
Heated scrambled peptide
Unheated corisin
Heated corisin
c
19 kDa
Cleaved caspase-3
46 kDa
βActin
62 kDa
p-Akt
62 kDa
t-Akt
Saline
Unheated scrambled peptide
Unheated corisin
Heated corisin
d
Cleaved caspase-3/βactin
e
p-Akt/t-Akt

Supplementary Figure 12. The synthetic pro-apoptotic peptide (corisin) is heat-stable. The synthetic corisin (5 μM; Peptide Institute Incorporation) or scrambled peptide (5 μM; Peptide Institute Incorporation) was incubated at 85ºC for 15 min before adding to the culture medium of A549 alveolar epithelial cells for 48h. **a**,**b**, Flow cytometry of A549 alveolar epithelial cells was performed after staining with propidium iodide and annexin V. Each treatment group with n=3. Bars indicate the means ± S.D. Statistical analysis by ANOVA with Newman-Keuls test. *$p<0.001$ vs unheated or heated scrambled peptide. **c**, In a separate experiment, The synthetic corisin (5 μM) or scrambled was incubated at 85ºC for 15 min before adding to the culture medium of A549 alveolar epithelial cells for 48h, and the cells were collected and prepared for Western blotting of cleaved caspase-3, βactin, total Akt (t-Akt), phosphorylated Akt (p-Akt). Each treatment group with n=3. Representative blot of each treatment group is shown. **d**,**e**, The intensity of the Western blot membrane bands was quantified by densitometry using the ImageJ software. Each treatment group with n=3. Bars indicate the means ± S.D. Statistical analysis by ANOVA with Newman-Keuls test. *$p<0.01$ vs saline. The source data underlying Supplementary Figs 12b,c,d,e are provided in the Source Data file.

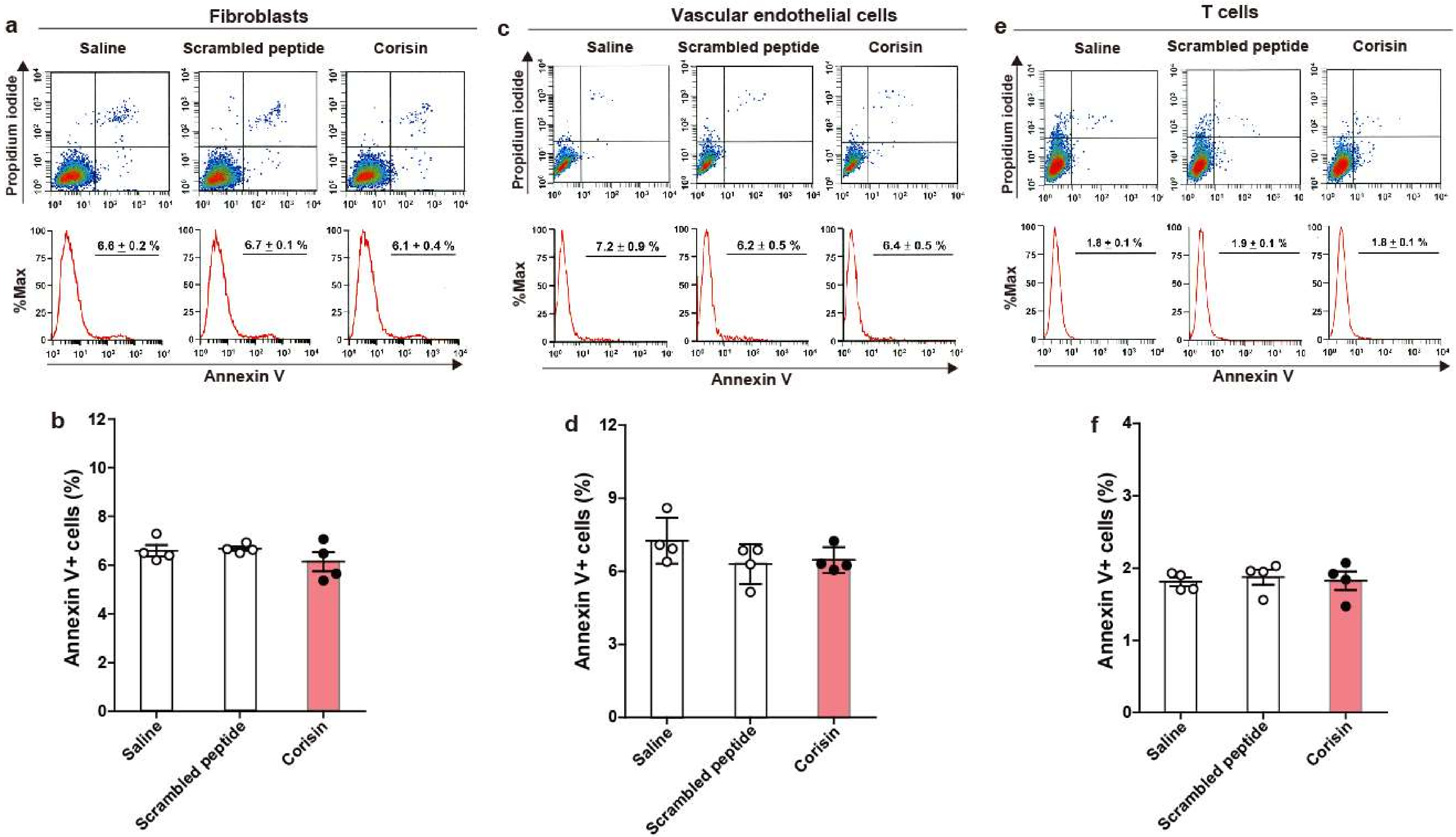
a
Fibroblasts
Saline
Scrambled peptide
Corisin
Propidium iodide
%Max
6.6 ± 0.2 %
6.7 ± 0.1 %
6.1 ± 0.4 %
Annexin V
c
Vascular endothelial cells
Saline
Scrambled peptide
Corisin
7.2 ± 0.9 %
6.2 ± 0.5 %
6.4 ± 0.5 %
Annexin V
e
T cells
Saline
Scrambled peptide
Corisin
1.8 ± 0.1 %
1.9 ± 0.1 %
1.8 ± 0.1 %
Annexin V
b
d
f
Annexin V+ cells (%)
Saline
Scrambled peptide
Corisin

Supplementary Figure 13. The apoptotic peptide (corisin) does not induce apoptosis of fibroblast, vascular endothelial cells or T cells. **a**,**b**, Flow cytometry analysis of HFL1 lung fibroblasts cultured for 48h in DMEM medium containing 10 μg/ml corisin. Each group with n=4. **c**,**d**, Flow cytometry analysis of human umbilical vein endothelial cells cultured for 48h in DMEM medium containing 10 μg/ml corisin. Each group with n=4. **e**,**f**, Flow cytometry analysis of human Jurkat T cells cultured for 48h in DMEM medium containing 10 μg/ml corisin. Each treatment group with n=4. Bars indicate the means ± S.D. Statistical analysis by ANOVA with Tukey’s test. The source data underlying Supplementary Figs 13b,d,f are provided in the Source Data file.

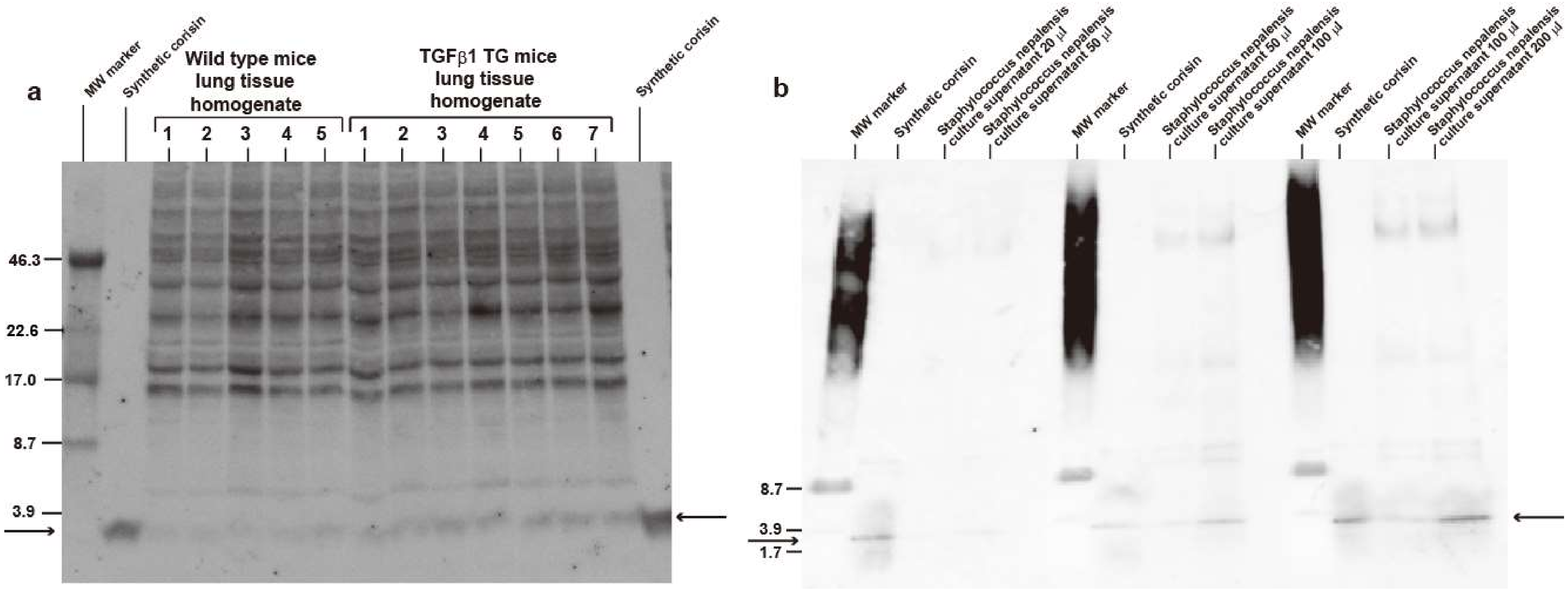

Supplementary Figure 14. A band at the corresponding molecular weight of corisin can be observed in Western blotting of mouse lung tissue samples and culture supernatant of *Staphylococcus nepalensis* using the corisin antibody. Five micrograms of lung tissue homogenate prepared from wild type and transforming growth factor (TGF)β1 transgenic (TG) mice (**a**), and several volumes of culture supernatant from *Staphylococcus nepalensis* (**b**) concentrated by precipitation with trichloroacetic acid were loaded on a 5-15% gradient sodium dodecyl sulfate polyacrylamide gel, and then Western blotting performed using anti-corisin antibody. Representative microphotographs out of two experiments with similar results are shown. Synthetic corisin was used as control. MW, molecular weight in kDa. Arrows indicate the band of corisin.

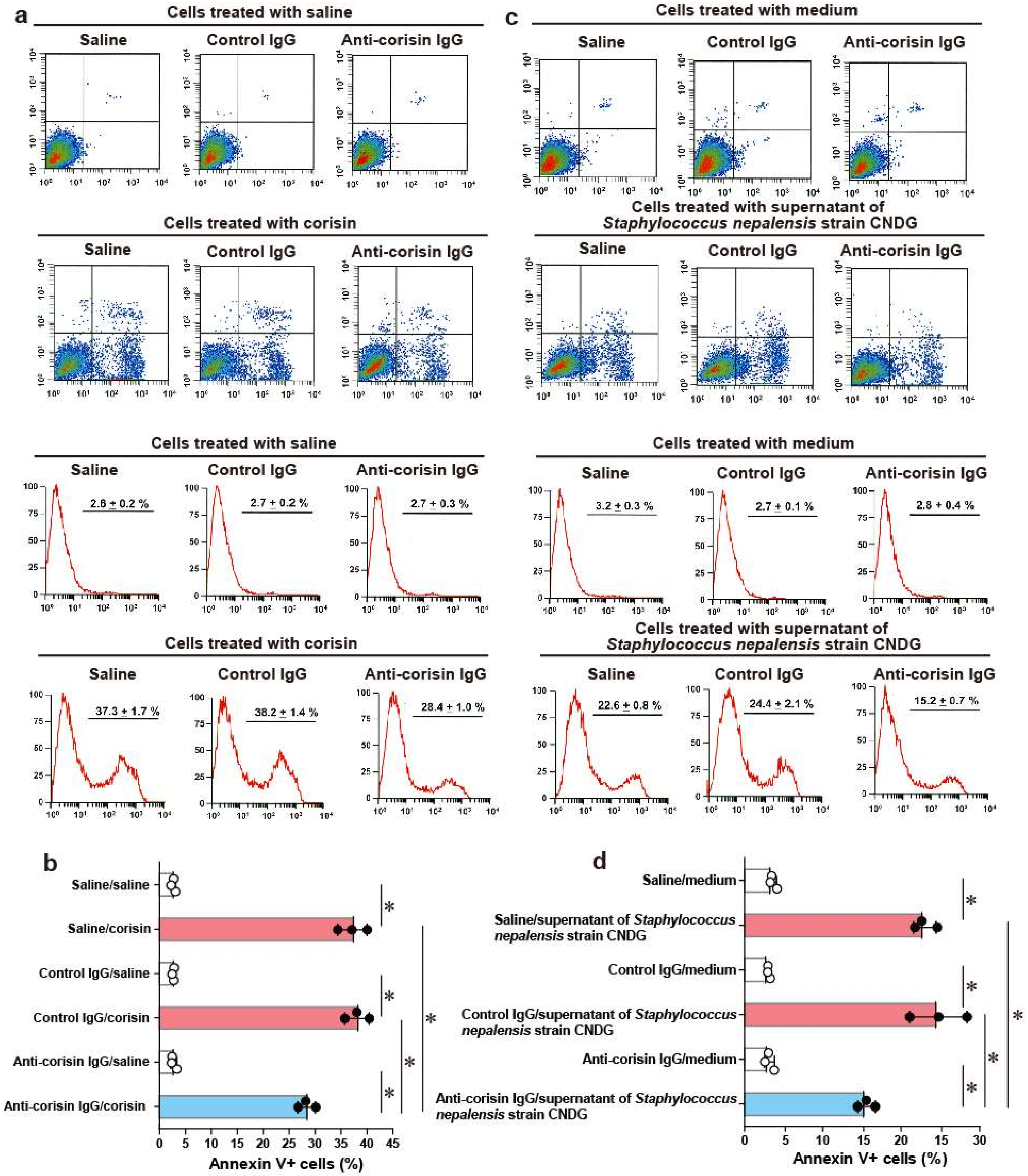

a
Cells treated with saline
Saline
Control IgG
Anti-corisin IgG
Cells treated with corisin
Cells treated with saline
2.6 ± 0.2 %
2.7 ± 0.2 %
2.7 ± 0.3 %
Cells treated with corisin
37.3 ± 1.7 %
38.2 ± 1.4 %
28.4 ± 1.0 %
c
Cells treated with medium
Cells treated with supernatant of *Staphylococcus nepalensis* strain CNDG
3.2 ± 0.3 %
2.7 ± 0.1 %
2.8 ± 0.4 %
22.6 ± 0.8 %
24.4 ± 2.1 %
15.2 ± 0.7 %
b
Saline/saline
Saline/corisin
Control IgG/saline
Control IgG/corisin
Anti-corisin IgG/saline
Anti-corisin IgG/corisin
Annexin V+ cells (%)
d
Saline/medium
Saline/supernatant of *Staphylococcus nepalensis* strain CNDG
Control IgG/medium
Control IgG/supernatant of *Staphylococcus nepalensis* strain CNDG
Anti-corisin IgG/medium
Anti-corisin IgG/supernatant of *Staphylococcus nepalensis* strain CNDG
Annexin V+ cells (%)

Supplementary Figure 15. Antibody against corisin inhibits the pro-apoptotic activity of corisin and of the supernatant of *Staphylococcus nepalensis* strain CNDG. **a**,**b**, A549 alveolar epithelial cells (2 x 10^5 cells/well) were cultured in 12-well plates and stimulated with 5 μM corisin in the presence of saline (Saline/corisin), 10 μg/ml control rabbit IgG (Control IgG/corisin) or 10 μg/ml rabbit anti-corisin IgG(Anti-corisin IgG/corisin) for 48h. Cells cultured in the presence of saline and treated with saline (Saline/saline), control rabbit IgG (Control IgG/saline) or rabbit ant-corisin IgG (Anti-corisin IgG/saline) were used as controls. Each treatment group with n=3 (triplicates). Bars indicate the means ± S.D. Statistical analysis by ANOVA with Tukey's test. *p<0.001. **c**,**d**, A549 alveolar epithelial cells cultured in 12-well plates were stimulated with the 1/10 dilution of the culture supernatant of *Staphylococcus nepalensis* strain CNDG in the presence of saline (Saline/supernatant of *Staphylococcus nepalensis* strain CNDG), 10 μg/ml control rabbit IgG (Control IgG/supernatant of *Staphylococcus nepalensis* strain CNDG) or 10 μg/ml rabbit anti-corisin IgG (Anti-corisin IgG/supernatant of *Staphylococcus nepalensis* strain CNDG) for 48h. Cells cultured in medium and treated with saline (Saline/medium), control rabbit IgG (Control IgG/medium) or rabbit ant-corisin IgG (Anti-corisin IgG/medium) were used as controls. Each treatment group with n=3. Flow cytometry of A549 cells was performed after staining with propidium iodide and annexin V. Bars indicate the means ± S.D. Statistical analysis by ANOVA with Tukey's test. *p<0.001. The source data underlying Supplementary Figs 15b,d are provided in the Source Data file.

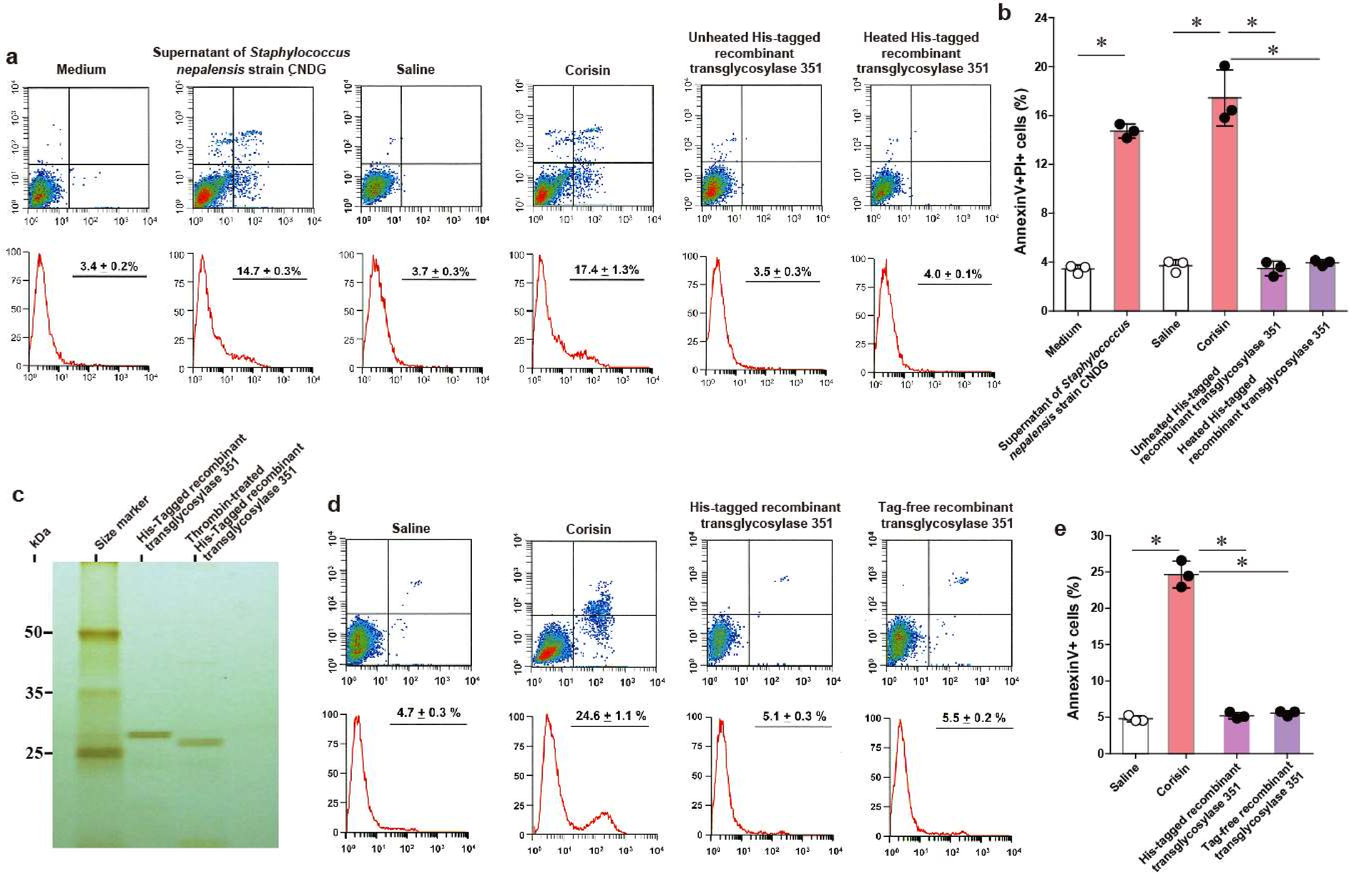
a
Medium
Supernatant of Staphylococcus nepalensis strain CNDG
Saline
Corisin
Unheated His-tagged recombinant transglycosylase 351
Heated His-tagged recombinant transglycosylase 351
3.4 ± 0.2%
14.7 ± 0.3%
3.7 ± 0.3%
17.4 ± 1.3%
3.5 ± 0.3%
4.0 ± 0.1%
b
AnnexinV+PI+ cells (%)
Medium
Supernatant of Staphylococcus nepalensis strain CNDG
Saline
Corisin
Unheated His-tagged recombinant transglycosylase 351
Heated His-tagged recombinant transglycosylase 351
c
kDa
Size marker
His-Tagged recombinant transglycosylase 351
Thrombin-treated His-Tagged recombinant transglycosylase 351
50
35
25
d
Saline
Corisin
His-tagged recombinant transglycosylase 351
Tag-free recombinant transglycosylase 351
4.7 ± 0.3 %
24.6 ± 1.1 %
5.1 ± 0.3 %
5.5 ± 0.2 %
e
AnnexinV+ cells (%)
Saline
Corisin
His-tagged recombinant transglycosylase 351
Tag-free recombinant transglycosylase 351

Supplementary Figure 16. The full-length transglycosylase 351 containing the corisin sequence has no apoptotic activity. a,**b**, Flow cytometry analysis of A549 alveolar epithelial cells cultured for 48h in DMEM medium containing 10 µg/ml corisin, unheated or heated His-tagged recombinant transglycosylase. Each treatment group with n=3. Bars indicate the means ± S.D. Statistical analysis by ANOVA with Tukey's test. *p<0.001. **c**, Electrophoresis using sodium dodecyl sulfate polyacrylamide gel (10-20%) and silver-staining of thrombin-treated or thrombin-untreated His-tagged recombinant transglycosylase 351 from *Staphylococcus nepalensis* strain CNDG. Representative microphotographs out of two experiments with similar results are shown. **d**,**e**, Flow cytometry analysis of A549 alveolar epithelial cells cultured for 48h in DMEM medium containing 10 µg/ml corisin, His-tagged or Tag-free recombinant transglycosylase. Each treatment group with n=3. Bars indicate the means ± S.D. Statistical analysis by ANOVA with Tukey's test. *p<0.001. The source data underlying Supplementary Figs 16b,e are provided in the Source Data file.

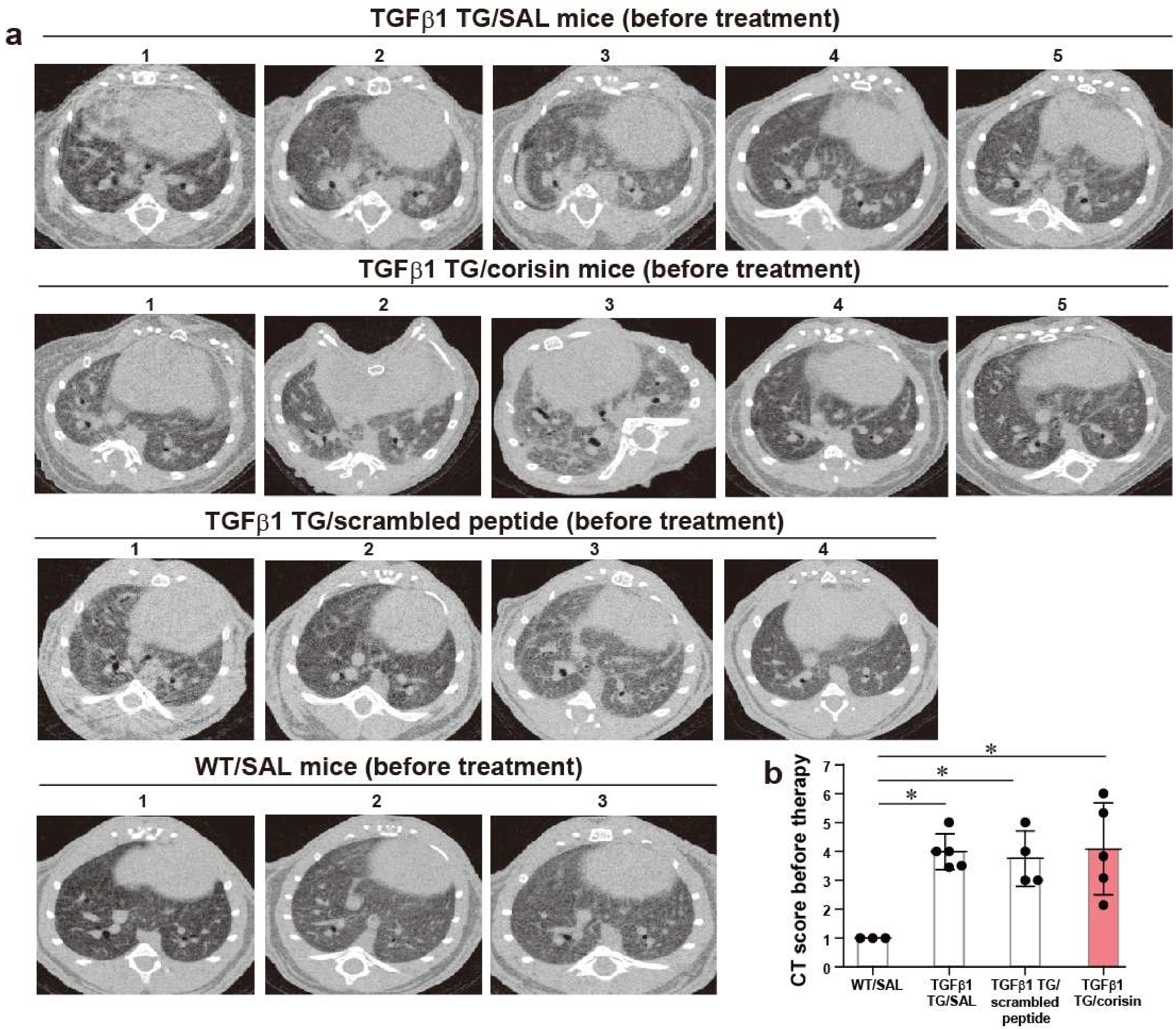
a
TGFβ1 TG/SAL mice (before treatment)
1
2
3
4
5
TGFβ1 TG/corisin mice (before treatment)
1
2
3
4
5
TGFβ1 TG/scrambled peptide (before treatment)
1
2
3
4
WT/SAL mice (before treatment)
1
2
3
b
CT score before therapy
0
1
2
3
4
5
6
7
*
*
*
WT/SAL
TGFβ1 TG/SAL
TGFβ1 TG/ scrambled peptide
TGFβ1 TG/corisin

Supplementary Figure 17. CT findings in mice used for intra-tracheal instillation of corisin, scrambled peptide or saline. a,**b**, Computed tomography (CT) and CT fibrosis scoring of wild-type (WT) mice (n=3) and transforming growth factor (TGF)β1 transgenic (TG) mice before treatment with saline (n=5), scrambled peptide (n=4) or corisin (n=5) were performed as described under methods. Bars indicate the means ± S.D. Statistical analysis by ANOVA with Tukey's test. *$p<0.05$. No statistical difference ($p=0.9$) between TGFβ1 TG/SAL, TGFβ1 TG/scrambled peptide, and TGFβ1 TG/corisin groups. The source data underlying Supplementary Fig 17b are provided in the Source Data file.

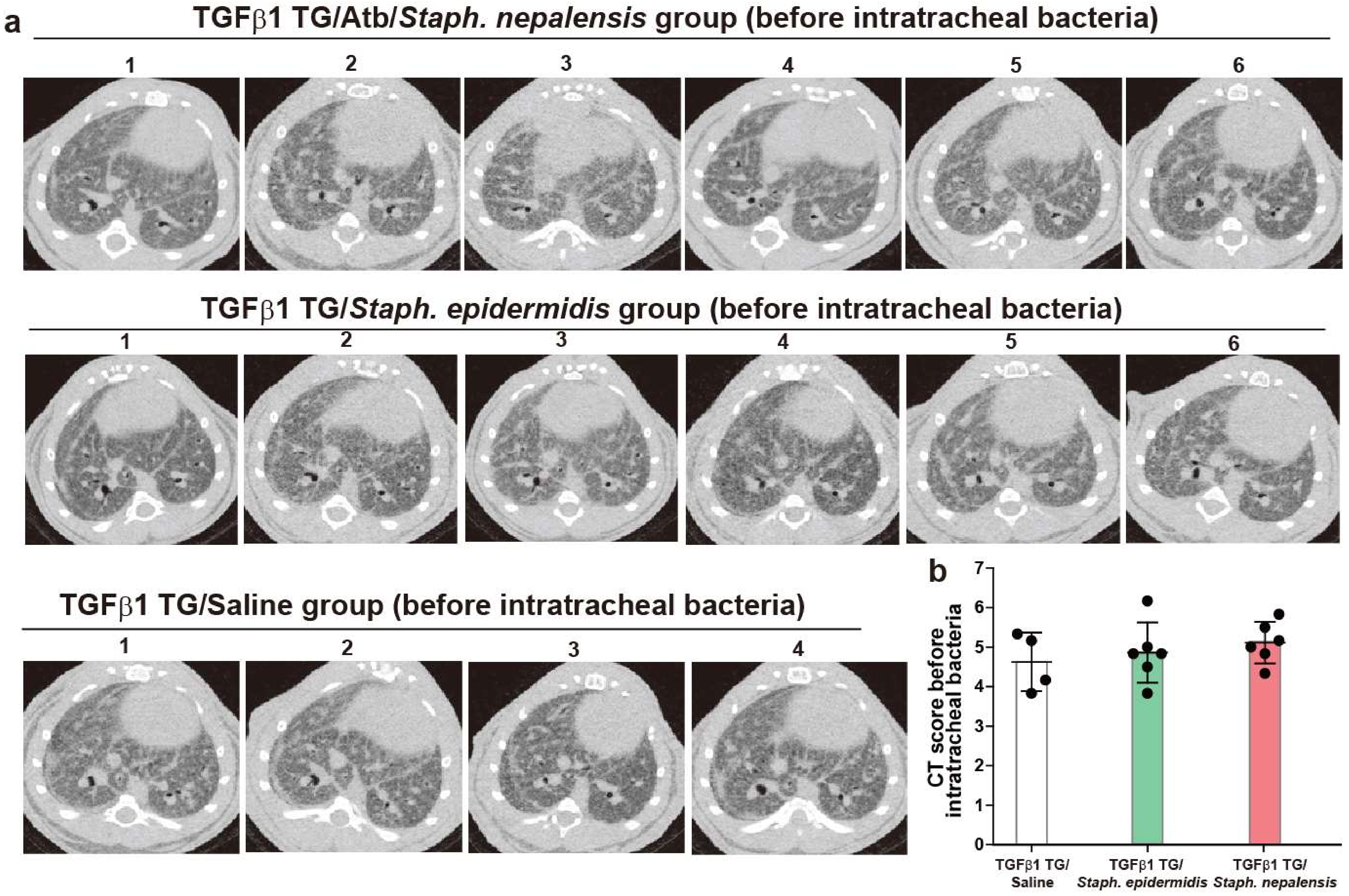
a
TGFβ1 TG/Atb/Staph. nepalensis group (before intratracheal bacteria)
1
2
3
4
5
6
TGFβ1 TG/Staph. epidermidis group (before intratracheal bacteria)
1
2
3
4
5
6
TGFβ1 TG/Saline group (before intratracheal bacteria)
1
2
3
4
b
CT score before intratracheal bacteria
0
1
2
3
4
5
6
7
TGFβ1 TG/ Saline
TGFβ1 TG/ Staph. epidermidis
TGFβ1 TG/ Staph. nepalensis

Supplementary Figure 18. CT findings in mice used for intra-tracheal instillation of *Staphylococcus nepalensis*, *Staphylococcus epidermidis* or saline. a,**b**, Computed tomography (CT) and CT fibrosis scoring of transforming growth factor (TGF)β1 transgenic (TG) mice before intra-tracheal instillation of *Staphylococcus nepalensis* (n=6), *Staphylococcus epidermidi*s (n=6) or saline (n=4) as described under methods. Bars indicate the means ± S.D. Statistical analysis by ANOVA with Tukey's test. No statistical difference (p=0.5) between all mouse groups The source data underlying Supplementary Fig 18b are provided in the Source Data file.

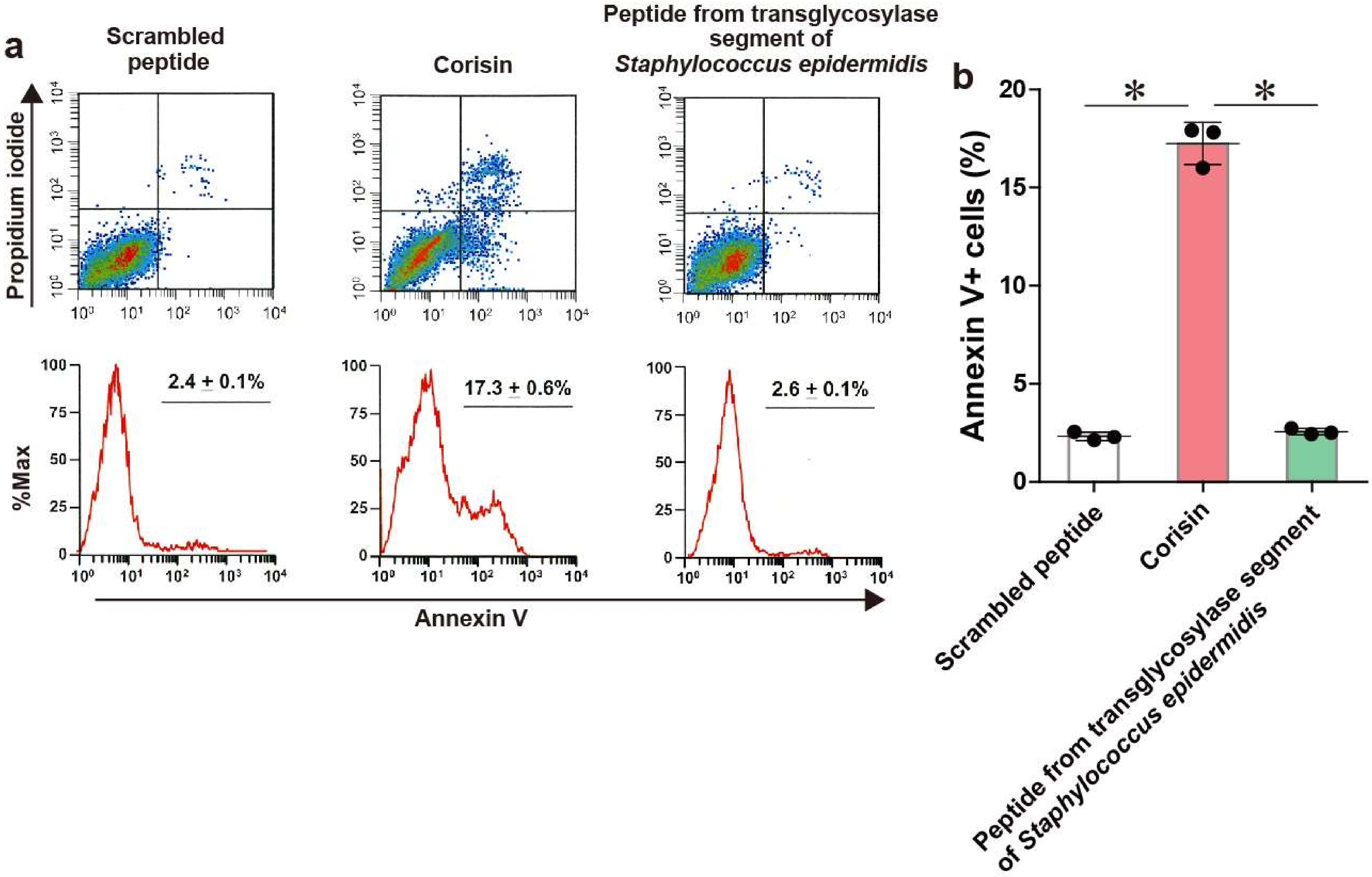
a
Scrambled peptide
Corisin
Peptide from transglycosylase segment of *Staphylococcus epidermidis*
Propidium iodide
%Max
2.4 ± 0.1%
17.3 ± 0.6%
2.6 ± 0.1%
Annexin V
b
Annexin V+ cells (%)
*
*
Scrambled peptide
Corisin
Peptide from transglycosylase segment of *Staphylococcus epidermidis*

Supplementary Figure 19. The synthetic peptide containing the sequence of the transglycosylase segment (corisin) from *Staphylococcus nepalensis* strain CNDG, but not its scrambled peptide or the synthetic peptide containing the sequence of the transglycosylase segment from Staphylococcus *epidermidis*, induces apoptosis of alveolar epithelial cells. a,**b**, Flow cytometry analysis of A549 alveolar epithelial cells cultured for 24h in DMEM medium containing 10 μM of synthetic peptide containing the sequence of the transglycosylase segment (corisin) from *Staphylococcus nepalensis* strain CNDG (IVMPESSGNPNAVNPAGYR), its scrambled peptide (NRVYNGPAASPVSEGMPIN) or synthetic peptide of the transglycosylase segment from *Staphylococcus epidermidis* (ATCC14990) (IIARESNGQLHARNASGAA). Each treatment group with n=3 (triplicates). Bars indicate the means ± S.D. Statistical analysis by ANOVA with Tukey's test. *p<0.001. The source data underlying Supplementary Fig 19b are provided in the Source Data file.

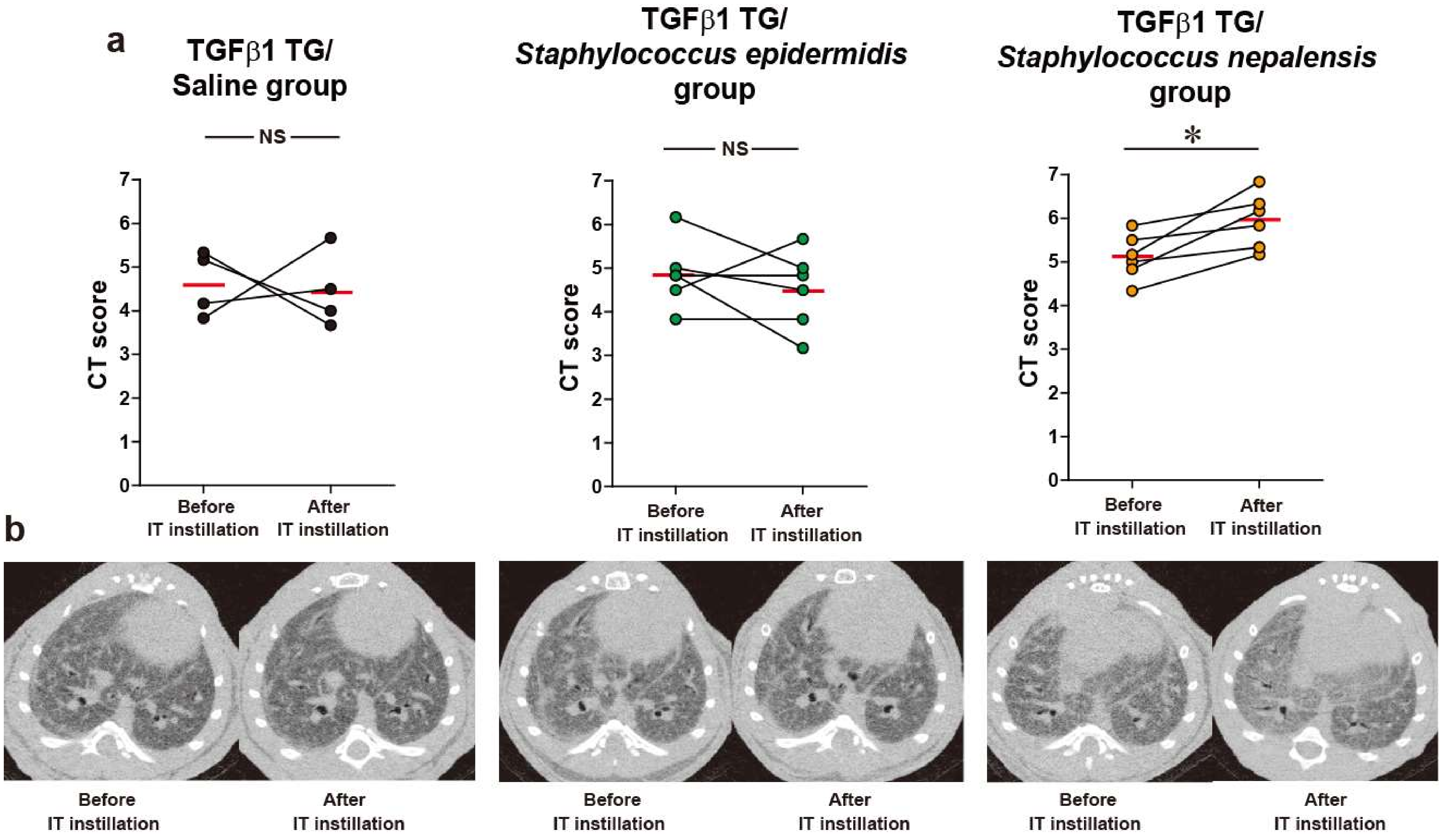

Supplementary Figure 20. Deterioration of radiological findings in germ-free TGFβ1 TG mice after intra-tracheal instillation of *Staphylococcus nepalensis*. a,**b**, Computed tomography (CT) and CT fibrosis scoring of transforming growth factor (TGF)β1 transgenic (TG) mice were performed before and after intra-tracheal instillation of saline (n=4), *Staphylococcus epidermidis* (n=6) or *Staphylococcus nepalensis* (n=6) in germ-free TGFβ1 TG mice as described under methods. Bars indicate the means ± S.D. Statistical analysis by two-tailed Mann-Whitney U test. *p<0.05. The source data underlying Supplementary Fig 20a are provided in the Source Data file.

Supplementary Table 3. IPF patients characteristics

Clinical parameters	No of patients and mean values
No of Japanese patients	34
Sex	
Male	29
Female	5
Age (years-old)	71.7 ± 6.6
Smoking history	
Current smoker	2
Ex-smoker	25
Never smoker	7
Lung function test	
VC (L)	2.7 ± 0.7
VC (% predicted)	80.8 ± 17.3
FVC (L)	2.7 ± 0.7
FVC (% predicted)	83.3 ± 18.4
FEV1 (L)	2.1 ± 0.6
FEV1/FVC (%)	78.8 ± 10.9
Rest SpO_2 (%)	95.6 ± 2.2
Therapy	
None	32
Nintedanib	2

Data are the mean ± S.D. IPF, idiopathic pulmonary fibrosis; VC, vital capacity; FEV1, forced expiratory volume in one second; FVC, forced volume vital capacity; L, liters; SpO_2, arterial oxygen saturation by pulse oximetry. Source data are provided in the Source Data file.

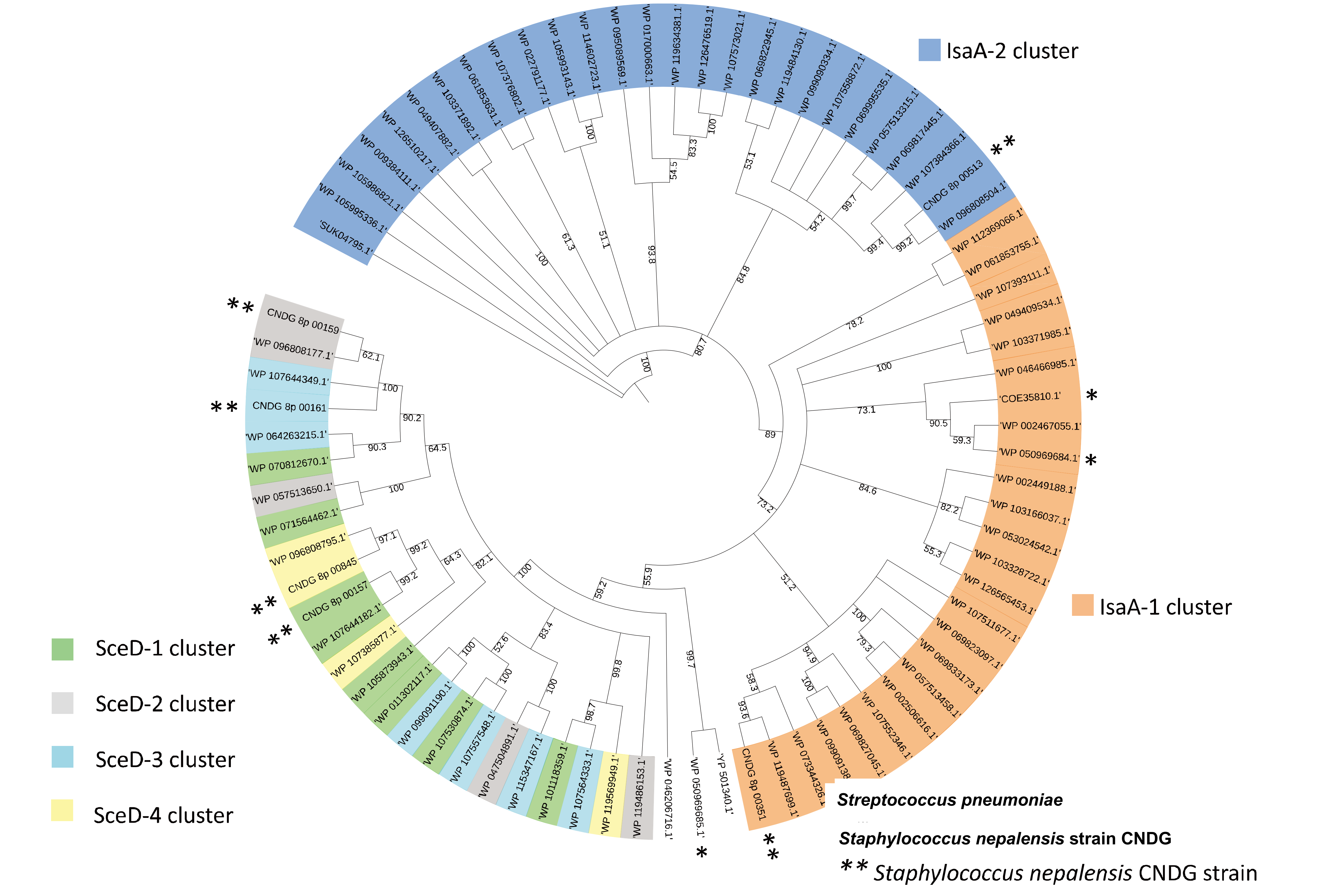

IsaA-2 cluster
IsaA-1 cluster
SceD-1 cluster
SceD-2 cluster
SceD-3 cluster
SceD-4 cluster
* Streptococcus pneumoniae
* Staphylococcus nepalensis strain CNDG
** Staphylococcus nepalensis CNDG strain
'SUK04795.1'
'WP 105995336.1'
'WP 105986821.1'
'WP 009384111.1'
'WP 126510217.1'
'WP 049407882.1'
'WP 103371892.1'
'WP 061853631.1'
'WP 107376802.1'
'WP 022791177.1'
'WP 105993143.1'
'WP 114602723.1'
'WP 050685569.1'
'WP 017000663.1'
'WP 119634381.1'
'WP 126476519.1'
'WP 107573021.1'
'WP 069822945.1'
'WP 119484130.1'
'WP 099090334.1'
'WP 107558872.1'
'WP 069995535.1'
'WP 057513315.1'
'WP 069817445.1'
'WP 107384366.1'
CNDG 8p 00513
'WP 096808504.1'
'WP 112369066.1'
'WP 061853755.1'
'WP 107393111.1'
'WP 049409534.1'
'WP 103371985.1'
'WP 046466985.1'
'COE35810.1'
'WP 002467055.1'
'WP 050969684.1'
'WP 002449188.1'
'WP 103166037.1'
'WP 053024542.1'
'WP 103328722.1'
'WP 126565453.1'
'WP 107511677.1'
'WP 069823097.1'
'WP 069833173.1'
'WP 057513458.1'
'WP 002506616.1'
'WP 107552346.1'
'WP 069827045.1'
'WP 119487699.1'
CNDG 8p 00351
'YP 501340.1'
'WP 050969685.1'
'WP 046206716.1'
'WP 119486153.1'
'WP 119569949.1'
'WP 107564333.1'
'WP 101118359.1'
'WP 115347167.1'
'WP 047504891.1'
'WP 107557548.1'
'WP 107530874.1'
'WP 099091190.1'
'WP 011302117.1'
'WP 105873943.1'
'WP 107385877.1'
'WP 107644182.1'
CNDG 8p 00157
CNDG 8p 00845
'WP 096808795.1'
'WP 071564462.1'
'WP 057513650.1'
'WP 070812670.1'
'WP 064263215.1'
CNDG 8p 00161
'WP 107644349.1'
'WP 096808177.1'
CNDG 8p 00159

Supplementary Figure 21. Phylogenetic analysis of the *Staphylococcus nepalensis* strain CNDG transglycosylases and their relatives in the genus *Staphylococcus*. A phylogenetic tree based on amino acid sequences aligned using MUSCLE alignment was constructed by the Neighbor joining method. Bootstraps were performed with 1,000 replicates. The GenBank accession numbers in this tree are as follows: **CLUSTER IsaA-1** ■ [WP_112369066.1 (transglycosylase, *S. arlettae*), WP_061853755.1 (hypothetical protein, *S. kloosii*), WP_107393111.1 (transglycosylase, *S. auricularis*), WP_049409534.1 (hypothetical protein, *S. pettenkoferi*), WP_103371985.1 (transglycosylase, *S. argensis*), WP_046466985.1 (transglycosylase, *S. pasteuri*), COE35810.1 (transglycosylase, *Streptococcus pneumoniae*), WP_002467055.1 (hypothetical protein, *S. warneri*), WP_050969684.1 (transglycosylase, *Streptococcus pneumoniae* type N), WP_002449188.1 (hypothetical protein, *S. hominis*), WP_103166037.1 (transglycosylase, *S. devriesei*), WP_053024542.1 (transglycosylase, *S. haemolyticus*), WP_103328722.1 (transglycosylase, *S. petrasii*), WP_126565453.1 (transglycosylase, *S. carnosus*), WP_107511677.1 (transglycosylase, *S. gallinarum*), WP_069823097.1 (transglycosylase, *S. succinus*), WP_069833173.1 (transglycosylase, *S. equorum*), WP_057513458.1 (hypothetical protein, *S.* sp. NAM3COL9), WP_002506616.1 (hypothetical protein, *S.* sp. OJ82), WP_107552346.1 (transglycosylase, *S. xylosus*), WP_069827045.1 (transglycosylase, *S. saprophyticus*), WP_099091381.1 (transglycosylase, *S. edaphicus*), WP_073344326.1 (transglycosylase, *S. cohnii*), WP_119487699.1 (transglycosylase, *S. nepalensis*), CNDG_8p_00351 (putative transglycosylase IsaA-1, *S. nepalensis*)] **CLUSTER IsaA-2** ■ [SUK04795.1 SceA (*S. aureus*), WP_105995336.1 (hypothetical protein, *S. agnetis*), WP_105986821.1 (hypothetical protein, *S. chromogenes*), WP_009384111.1 (hypothetical protein, *S. massiliensis*), WP_126510217.1 (transglycosylase, *S. epidermidis*), WP_049407882.1 (hypothetical protein, *S. pettenkoferi*), WP_103371892.1 (hypothetical protein, *S. argensis*), WP_061853631.1 (hypothetical protein, *S. kloosii*), WP_107376802.1 (hypothetical protein, *S. arlettae*), WP_022791177.1 LysM peptidoglycan-binding domain-containing protein (*Weissella halotolerans*), WP_105993143.1 (hypothetical protein, *S. simulans*), WP_114602723.1 (hypothetical protein, *S.* sp. EZ-P03), WP_095089569.1 (hypothetical protein, *S. stepanovicii*), WP_017000663.1 (hypothetical protein, *S. lentus*), WP_119634381.1 (hypothetical protein, *S. fleurettii*), WP_126476519.1 (hypothetical protein, *S. schleiferi*), WP_107573021.1 (hypothetical protein, *S. sciuri*), WP_069822945.1 (hypothetical protein, *S. succinus*), WP_119484130.1 (hypothetical protein, *S. gallinarum*), WP_099090334.1 (hypothetical protein, *S. edaphicus*), WP_107558872.1 (hypothetical protein, *S. xylosus*), WP_069995535.1 (hypothetical protein, *S. saprophyticus*), WP_057513315.1 (hypothetical protein, *S. sp.* NAM3COL9), WP_069817445.1 (hypothetical protein, *S. equorum*), WP_107384366.1 (hypothetical protein, *S. cohnii*), CNDG_8p_00513 (putative transglycosylase IsaA-2, *S. nepalensis*), WP_096808504.1 (hypothetical protein, *S. nepalensis*)] **CLUSTER SceD-1** ■ [WP_101118359.1 (transglycosylase, *S. succinus*), WP_107530874.1 (transglycosylase, *S. xylosus*), WP_011302117.1 transglycosylase SceD 1 (*S. saprophyticus*), WP_105873943.1 (transglycosylase, *S. cohnii*), WP_107644182.1 (transglycosylase, *S. nepalensis*), CNDG_8p_00157 (putative transglycosylase SceD-1, *S. nepalensis*), WP_071564462.1 (transglycosylase, *S. equorum*)] **CLUSTER SceD-2** ■ [WP_070812670.1 (transglycosylase, *S.* sp. HMSC034G07), WP_119486153.1 (transglycosylase, *S. gallinarum*), WP_047504891.1 (transglycosylase, *S.* sp. ZWU0021), WP_057513650.1 (transglycosylase, *S.* sp. NAM3COL9), WP_096808177.1 (transglycosylase, *S. nepalensis*), CNDG_8p_00159 (putative transglycosylase SceD-2, *S. nepalensis*)] **CLUSTER SceD-3** ■ [WP_107564333.1 (transglycosylase, *S. succinus*), WP_115347167.1 (transglycosylase, *S. saprophyticus*), WP_107557548.1 (transglycosylase, *S. xylosus*), WP_099091190.1 (transglycosylase, *S. edaphicus*), WP_064263215.1 (transglycosylase, *S. cohnii*), CNDG_8p_00161 (putative transglycosylase SceD-3, *S. nepalensis*), WP_107644349.1 (transglycosylase, *S. nepalensis*)] **CLUSTER SceD-4** ■ [WP_119569949.1 (transglycosylase, *S. succinus*), WP_107385877.1 (transglycosylase, *S. cohnii*), CNDG_8p_00845 (putative transglycosylase SceD-4, *S. nepalensis*) , WP_096808795.1 (transglycosylase, *S. nepalensis*)]. WP_050969685.1 (transglycosylase, *Streptococcus pneumoniae* type N), YP_501340.1 (transglycosylase, *S. aureus* subsp. *aureus* NCTC 8325), WP_046206716.1 (transglycosylase, *S. cohnii* subs. *cohnii*).

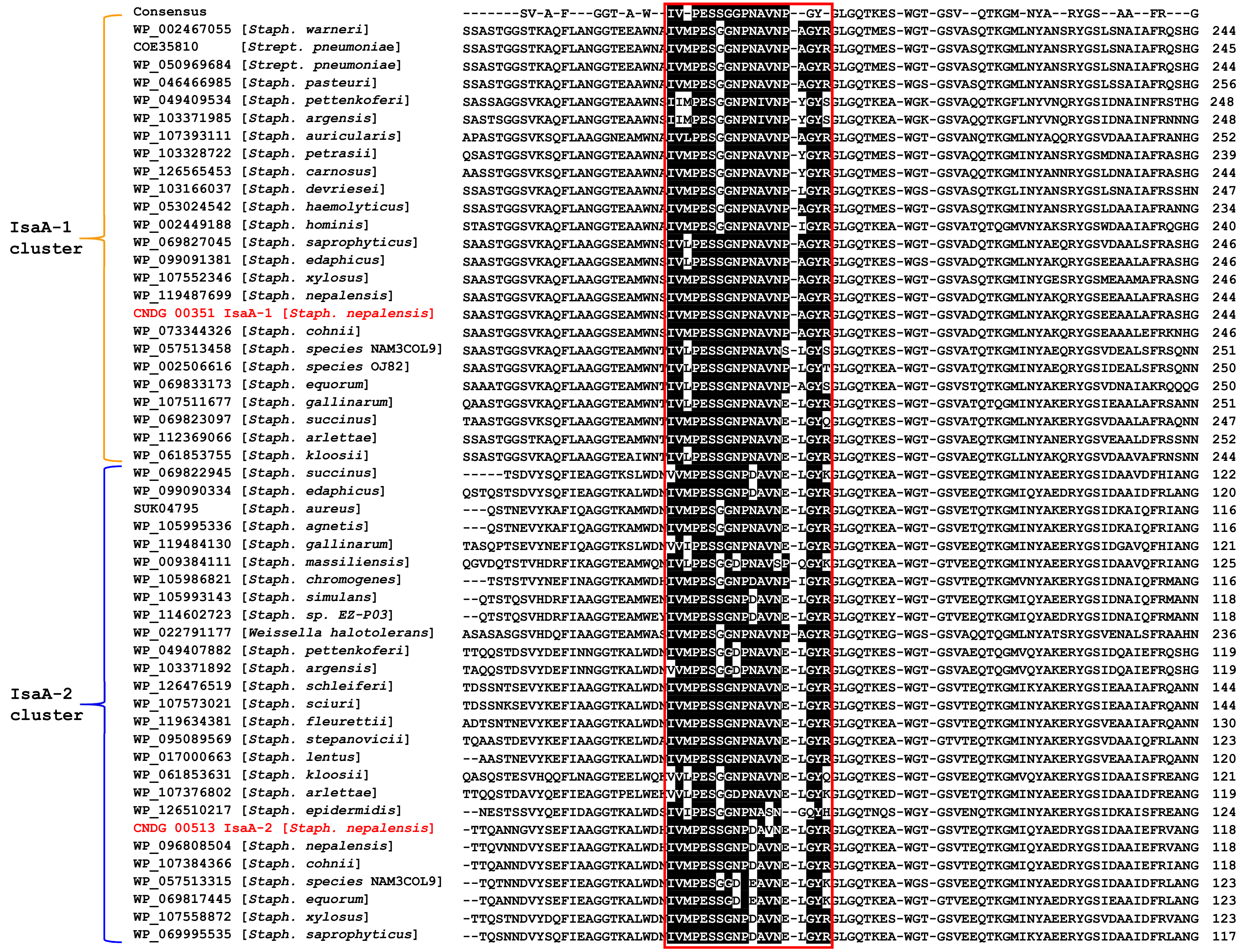

IsaA-1 cluster
IsaA-2 cluster
Consensus
WP_002467055 [Staph. warneri]
COE35810 [Strept. pneumoniae]
WP_050969684 [Strept. pneumoniae]
WP_046466985 [Staph. pasteuri]
WP_049409534 [Staph. pettenkoferi]
WP_103371985 [Staph. argensis]
WP_107393111 [Staph. auricularis]
WP_103328722 [Staph. petrasii]
WP_126565453 [Staph. carnosus]
WP_103166037 [Staph. devriesei]
WP_053024542 [Staph. haemolyticus]
WP_002449188 [Staph. hominis]
WP_069827045 [Staph. saprophyticus]
WP_099091381 [Staph. edaphicus]
WP_107552346 [Staph. xylosus]
WP_119487699 [Staph. nepalensis]
CNDG 00351 IsaA-1 [Staph. nepalensis]
WP_073344326 [Staph. cohnii]
WP_057513458 [Staph. species NAM3COL9]
WP_002506616 [Staph. species OJ82]
WP_069833173 [Staph. equorum]
WP_107511677 [Staph. gallinarum]
WP_069823097 [Staph. succinus]
WP_112369066 [Staph. arlettae]
WP_061853755 [Staph. kloosii]
WP_069822945 [Staph. succinus]
WP_099090334 [Staph. edaphicus]
SUK04795 [Staph. aureus]
WP_105995336 [Staph. agnetis]
WP_119484130 [Staph. gallinarum]
WP_009384111 [Staph. massiliensis]
WP_105986821 [Staph. chromogenes]
WP_105993143 [Staph. simulans]
WP_114602723 [Staph. sp. EZ-P03]
WP_022791177 [Weissella halotolerans]
WP_049407882 [Staph. pettenkoferi]
WP_103371892 [Staph. argensis]
WP_126476519 [Staph. schleiferi]
WP_107573021 [Staph. sciuri]
WP_119634381 [Staph. fleurettii]
WP_095089569 [Staph. stepanovicii]
WP_017000663 [Staph. lentus]
WP_061853631 [Staph. kloosii]
WP_107376802 [Staph. arlettae]
WP_126510217 [Staph. epidermidis]
CNDG 00513 IsaA-2 [Staph. nepalensis]
WP_096808504 [Staph. nepalensis]
WP_107384366 [Staph. cohnii]
WP_057513315 [Staph. species NAM3COL9]
WP_069817445 [Staph. equorum]
WP_107558872 [Staph. xylosus]
WP_069995535 [Staph. saprophyticus]

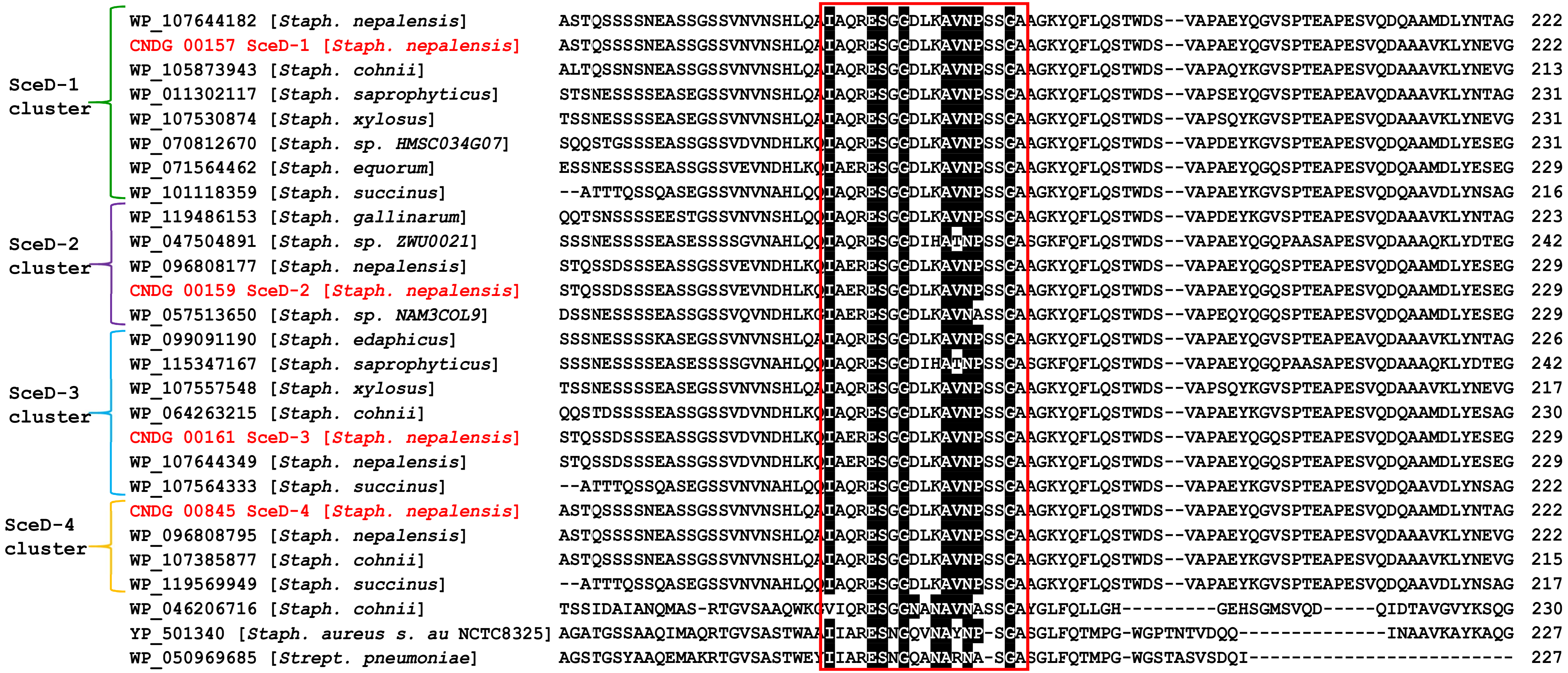

Supplementary Figure 22. Multiple sequence alignment showing conserved sequence of the pro-apoptotic segment of transglycosylases in several species of *Staphylococcus* and *Streptococcus*. The red square encloses sequences of the pro-apoptotic segment (tested and predicted). The multiple transglycosylases of *Staphylococcus nepalensis* strain CNDG are highlighted in red. Transglycosylases are grouped in several clusters based on transglycosylases encoded in *Staphylococcus nepalensis* strain CNDG. The amino acids that are identical (shaded black) to amino acids of the pro-apoptotic segment are indicated.

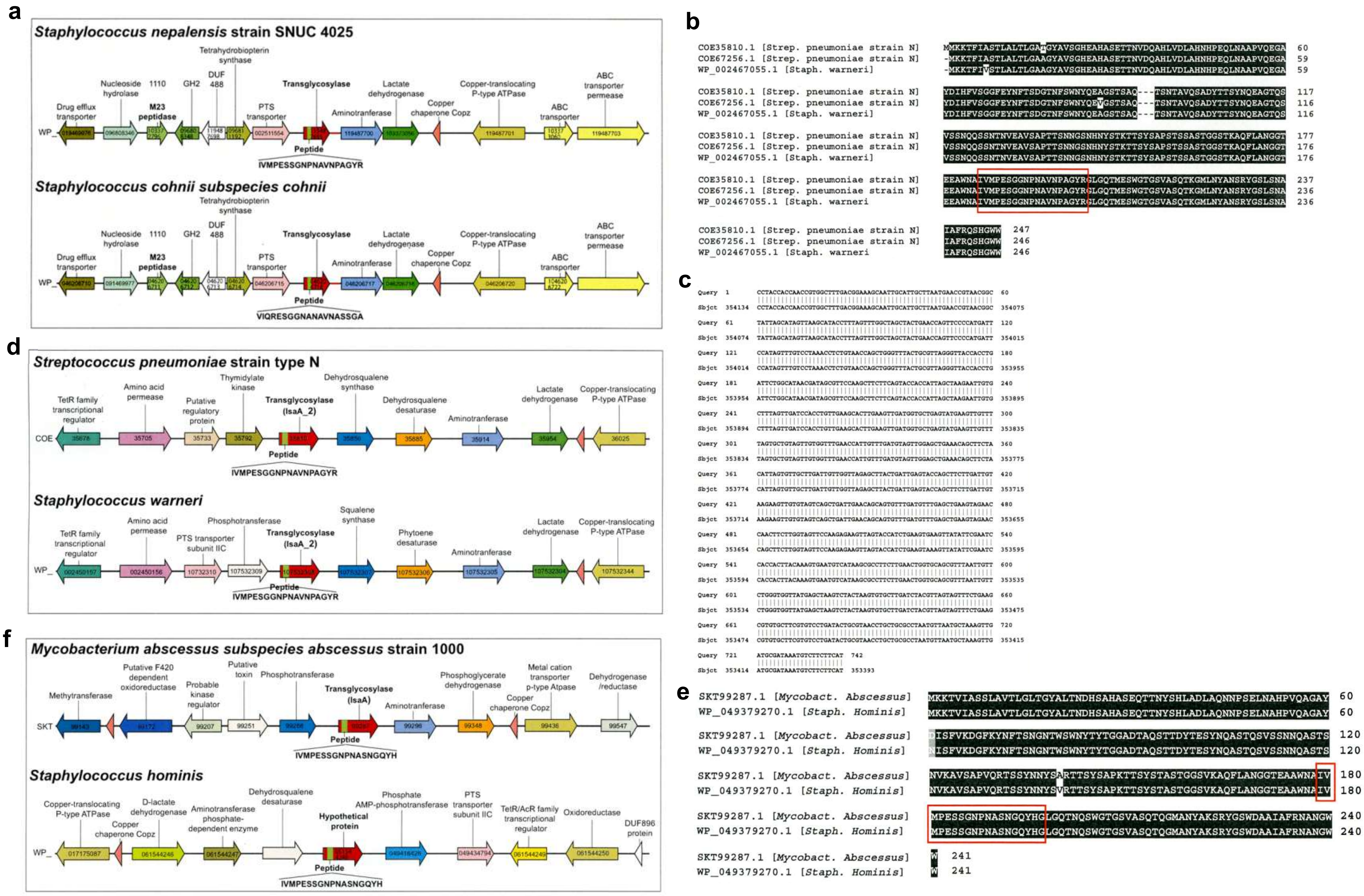
a
Staphylococcus nepalensis strain SNUC 4025
Drug efflux transporter
Nucleoside hydrolase
1110
M23 peptidase
GH2
Tetrahydrobiopterin synthase
DUF 488
PTS transporter
Transglycosylase
Aminotranferase
Lactate dehydrogenase
Copper chaperone Copz
Copper-translocating P-type ATPase
ABC transporter
ABC transporter permease
WP_
Peptide
IVMPESSGNPNAVNPAGYR
Staphylococcus cohnii subspecies cohnii
Drug efflux transporter
Nucleoside hydrolase
1110
M23 peptidase
GH2
Tetrahydrobiopterin synthase
DUF 488
PTS transporter
Transglycosylase
Aminotranferase
Lactate dehydrogenase
Copper chaperone Copz
Copper-translocating P-type ATPase
ABC transporter
ABC transporter permease
WP_
Peptide
VIQRESGGNANAVNASSGA
b
COE35810.1 [Strep. pneumoniae strain N] MMKKTFIASTLALTLGAAGYAVSGHEAHASETTNVDQAHLVDLAHNHPEQLNAAPVQEGA 60
COE67256.1 [Strep. pneumoniae strain N] -MKKTFIASTLALTLGAAGYAVSGHEAHASETTNVDQAHLVDLAHNHPEQLNAAPVQEGA 59
WP_002467055.1 [Staph. warneri] -MKKTFIVSTLALTLGAAGYAVSGHEAHASETTNVDQAHLVDLAHNHPEQLNAAPVQEGA 59
COE35810.1 [Strep. pneumoniae strain N] YDIHFVSGGFEYNFTSDGTNFSWNYQEAGSTSAQ---TSNTAVQSADYTTSYNQEAGTQS 117
COE67256.1 [Strep. pneumoniae strain N] YDIHFVSGGFEYNFTSDGTNFSWNYQEVGSTSAQ---TSNTAVQSADYTTSYNQEAGTQS 116
WP_002467055.1 [Staph. warneri] YDIHFVSGGFEYNFTSDGTNFSWNYQEAGSTSAQ---TSNTAVQSADYTTSYNQEAGTQS 116
COE35810.1 [Strep. pneumoniae strain N] VSSNQQSSNTNVEAVSAPTTSNNGSNHNYSTKTTSYSAPSTSSASTGGSTKAQFLANGGT 177
COE67256.1 [Strep. pneumoniae strain N] VSSNQQSSNTNVEAVSAPTTSNNGSNHNYSTKTTSYSAPSTSSASTGGSTKAQFLANGGT 176
WP_002467055.1 [Staph. warneri] VSSNQQSSNTNVEAVSAPTTSNNGSNHNYSTKTTSYSAPSTSSASTGGSTKAQFLANGGT 176
COE35810.1 [Strep. pneumoniae strain N] EEAWNAIVMPESGGNPNAVNPAGYRGLGQTMESWGTGSVASQTKGMLNYANSRYGSLSNA 237
COE67256.1 [Strep. pneumoniae strain N] EEAWNAIVMPESGGNPNAVNPAGYRGLGQTMESWGTGSVASQTKGMLNYANSRYGSLSNA 236
WP_002467055.1 [Staph. warneri EEAWNAIVMPESGGNPNAVNPAGYRGLGQTMESWGTGSVASQTKGMLNYANSRYGSLSNA 236
COE35810.1 [Strep. pneumoniae strain N] IAFRQSHGWW 247
COE67256.1 [Strep. pneumoniae strain N] IAFRQSHGWW 246
WP_002467055.1 [Staph. warneri IAFRQSHGWW 246
c
d
Streptococcus pneumoniae strain type N
TetR family transcriptional regulator
Amino acid permease
Putative regulatory protein
Thymidylate kinase
Transglycosylase (IsaA_2)
Dehydrosqualene synthase
Dehydrosqualene desaturase
Aminotranferase
Lactate dehydrogenase
Copper-translocating P-type ATPase
COE
Peptide
IVMPESGGNPNAVNPAGYR
Staphylococcus warneri
TetR family transcriptional regulator
Amino acid permease
PTS transporter subunit IIC
Phosphotransferase
Transglycosylase (IsaA_2)
Squalene synthase
Phytoene desaturase
Aminotranferase
Lactate dehydrogenase
Copper-translocating P-type ATPase
WP_
Peptide
IVMPESGGNPNAVNPAGYR
e
SKT99287.1 [Mycobact. Abscessus] MKKTVIASSLAVTLGLTGYALTNDHSAHASEQTTNYSHLADLAQNNPSELNAHPVQAGAY 60
WP_049379270.1 [Staph. Hominis] MKKTVIASSLAVTLGLTGYALTNDHSAHASEQTTNYSHLADLAQNNPSELNAHPVQAGAY 60
SKT99287.1 [Mycobact. Abscessus] DISFVKDGFKYNFTSNGNTWSWNYTYTGGADTAQSTTDYTESYNQASTQSVSSNNQASTS 120
WP_049379270.1 [Staph. Hominis] NISFVKDGFKYNFTSNGNTWSWNYTYTGGADTAQSTTDYTESYNQASTQSVSSNNQASTS 120
SKT99287.1 [Mycobact. Abscessus] NVKAVSAPVQRTSSYNNYSARTTSYSAPKTTSYSTASTGGSVKAQFLANGGTEAAWNAIV 180
WP_049379270.1 [Staph. Hominis] NVKAVSAPVQRTSSYNNYSVRTTSYSAPKTTSYSTASTGGSVKAQFLANGGTEAAWNAIV 180
SKT99287.1 [Mycobact. Abscessus] MPESSGNPNASNGQYHGLGQTNQSWGTGSVASQTQGMANYAKSRYGSWDAAIAFRNANGW 240
WP_049379270.1 [Staph. Hominis] MPESSGNPNASNGQYHGLGQTNQSWGTGSVASQTQGMANYAKSRYGSWDAAIAFRNANGW 240
SKT99287.1 [Mycobact. Abscessus] W 241
WP_049379270.1 [Staph. Hominis] W 241
f
Mycobacterium abscessus subspecies abscessus strain 1000
Methytransferase
Putative F420 dependent oxidoreductase
Probable kinase regulator
Putative toxin
Phosphotransferase
Transglycosylase (IsaA)
Aminotranferase
Phosphoglycerate dehydrogenase
Copper chaperone Copz
Metal cation transporter p-type Atpase
Dehydrogenase /reductase
SKT
Peptide
IVMPESSGNPNASNGQYH
Staphylococcus hominis
Copper-translocating P-type ATPase
Copper chaperone Copz
D-lactate dehydrogenase
Aminotransferase phosphate-dependent enzyme
Dehydrosqualene desaturase
Hypothetical protein
Phosphate AMP-phosphotransferase
PTS transporter subunit IIC
TetR/AcR family transcriptional regulator
Oxidoreductase
DUF896 protein
WP_
Peptide
IVMPESSGNPNASNGQYH

Supplementary Figure 23. Genomic context and multiple sequence alignment showing conserved sequence of the pro-apoptotic segment of transglycosylases in several species of *Staphylococcus* and *Streptococcus*. a, Genomic context of transglycosylases containing the peptide “IVMPESSGNPNAVNPAGYR” or its derivative in *Staphylococcus nepalensis* strain SNUC 4025 and *Staphylococcus cohnii* subspecies *cohnii.* **b**, *Streptococcus pneumoniae* contains transglycosylases (COE35810 and COE67256) with almost identical peptide sequence to corisin. **c**, The query sequence and the subject sequence in the alignment are from *S. pneumoniae* strain N and *S. warneri*, respectively. The complementary nucleotide sequence encodes COE67256 and highly identical proteins in *Staphylococcus warneri* strain SWO, strain SGI, strain NCTC11044, strain NCTC7291, and strain 22.1. **d**, Genomic context of transglycosylases containing the corisin sequence or its derivative in *Streptococcus pneumoniae* strain N and *Staphylococcus warneri*. **e**, The genome of a strain of the emerging pathogen *Mycobacterium* [*Mycobacteroides*] *abscessus* harbors a transglycosylase (SKT99287) that is almost identical to a transglycosylase (WP_049379270) in *Staphylococcus hominis*. **f**, Genomic context of transglycosylases containing the corisin sequence or its derivative in *Mycobacterium* [*Mycobacteroides*] *abscessus* and *Staphylococcus hominis.* The red square encloses sequences with high identity to the pro-apoptotic segment identified in *Staphylococcus nepalensis* strain CNDG. The amino acids that are identical (shaded black) or similar (grey) are indicated. The genomic context of *Staphylococcus nepalensis* strain SNUC is generally conserved in *Staphylococcus nepalensis* strain CNDG. The grouping shows genomic context and potential for horizontal gene transfer in the non-staphylococcal strains. The original annotations in individual genomes are maintained.

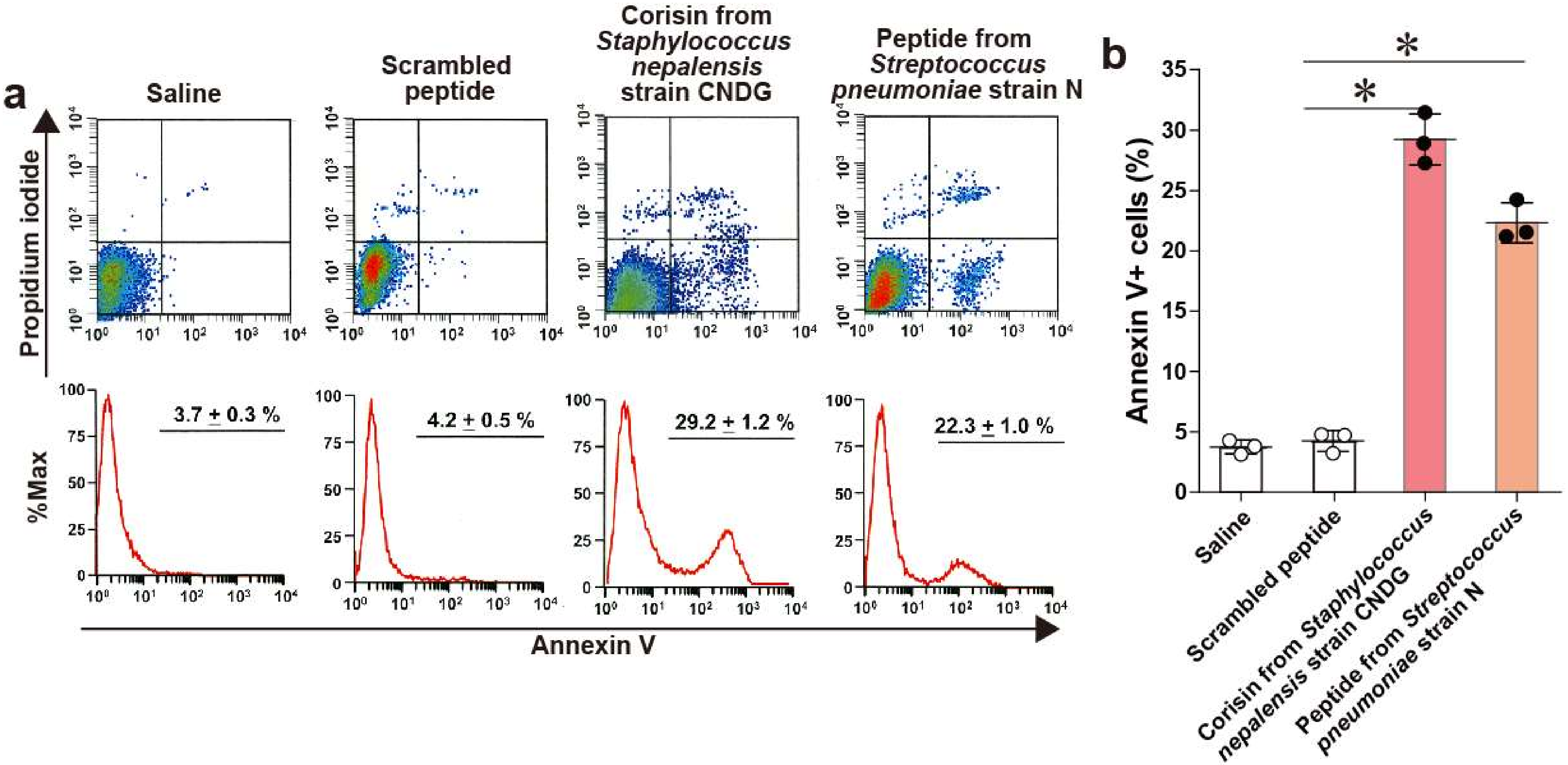

Supplementary Figure 24. The synthetic peptide from *Streptococcus pneumoniae* strain N transglycosylase has pro-apoptotic activity. a,**b**, Flow cytometry analysis of A549 alveolar cells cultured for 48h in DMEM medium containing 5 µM of the synthetic corisin (IVMPESSGNPNAVNPAGYR) from *Staphylococcus nepalensis* (strain CNDG) transglycosylase 351, its scrambled peptide (NRVYNGPAASPVSEGMPIN) or the synthetic peptide (IVMPESGGNPNAVNPAGYR) from *Streptococcus pneumoniae* strain N transglycosylases (COE35810 and COE6725). Each group with n=3. Bars indicate the means ± S.D. Statistical analysis by ANOVA with Tukey's test. *p<0.001. The source data underlying Supplementary Fig 24b are provided in the Source Data file.

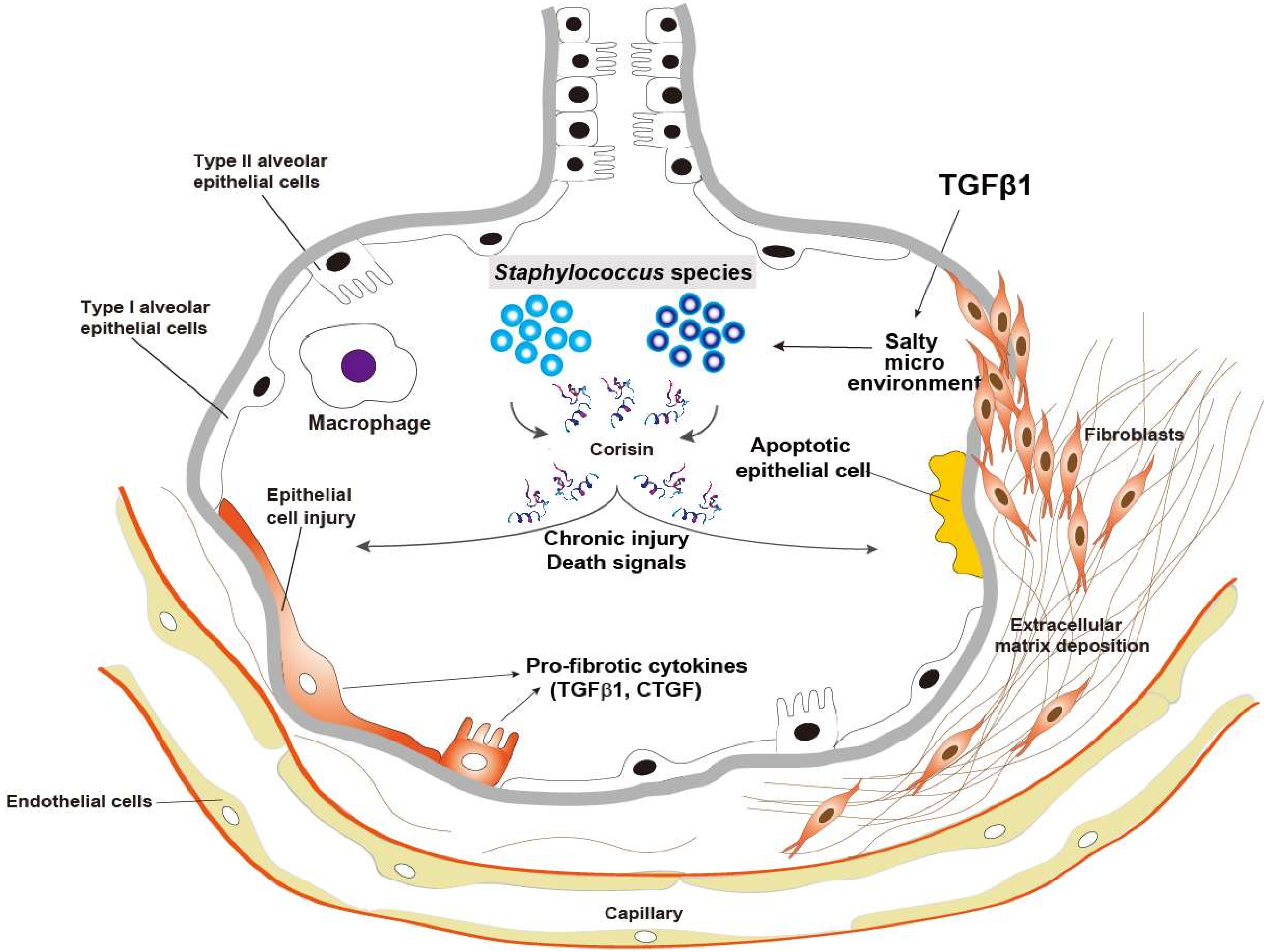
Type II alveolar epithelial cells
Type I alveolar epithelial cells
Staphylococcus species
TGFβ1
Salty micro environment
Macrophage
Corisin
Apoptotic epithelial cell
Fibroblasts
Epithelial cell injury
Chronic injury
Death signals
Extracellular matrix deposition
Pro-fibrotic cytokines (TGFβ1, CTGF)
Endothelial cells
Capillary

Supplementary Figure 25. Contribution of corisin to the pathogenesis of idiopathic pulmonary fibrosis. Transforming growth factor (TGF)β1 may increase the extracellular salt concentration by downregulating the cell surface expression of ion transporters, and the salty microenvironment stimulates the growth of *Staphylococcus* spp. that release corisin to induce apoptosis of alveolar epithelial cells. Excessive apoptosis and/or activation of epithelial cells contribute to acute exacerbation of pulmonary fibrosis.

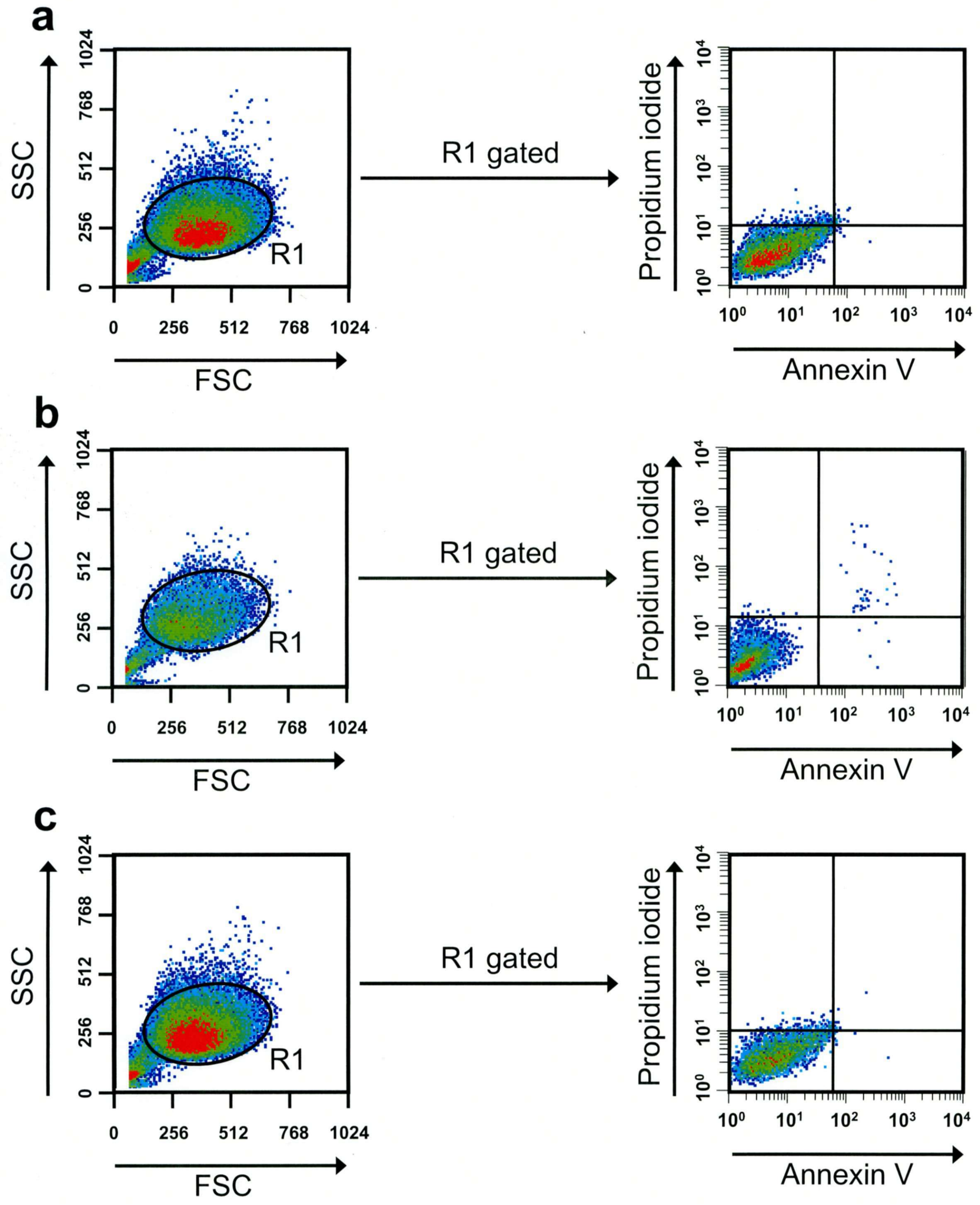

Supplementary Figure 26. Flow cytometry gating strategy. Gating strategies used in the experiments described in Supplementary Figure 4a (**a**), Supplementary Figure 11a (**b**), and Supplementary Figure 12a (**c**). Abbreviations: SSC, side scatter; FSC, forward scatter.

Supplementary Table 4. Antibodies used for flow cytometry

Target	Label	Clone	Source	Isotype	Company
Mouse Ly-6G/Ly-6C	FITC	RB6-8C5	rat	IgG2bκ	BioLegend, Inc. (San Diego, CA)
Mouse F4/80	PE	CIA3-1	rat	IgG2bκ	BioLegend, Inc. (San Diego, CA)
Mouse CD11c	PE/Cy5	N418	hamster	IgG	BioLegend, Inc. (San Diego, CA)
Mouse CD3ε	FITC	145-2C11	hamster	IgG	BioLegend, Inc. (San Diego, CA)
Mouse CD45R/B220	PE/Cy5	RA3-6B2	rat	IgG2aκ	BioLegend, Inc. (San Diego, CA)
Anti-mouse CD25	FITC	PC61	rat	IgG1λ	BioLegend, Inc. (San Diego, CA)
Mouse CD8a	PE	53-6.7	rat	IgG2aκ	BioLegend, Inc. (San Diego, CA)
mouse CD4	PE/Cy5	GK1.5	rat	IgG2bκ	BioLegend, Inc. (San Diego, CA)
mouse NK1.1	PE	PK136	mouse	IgG2aκ	BioLegend, Inc. (San Diego, CA)
Annexin V	FITC	-	-	-	BD Pharmingen (San Diego, CA)

FITC, fluorescein isothiocyanate; PE, phycoerythrin.

Supplementary Table 5. Primers for RT-PCR

	Sequence (5' -> 3')	Tm	Reference	Location	Product size
Ctfr					
Sense	CACAGTCATCAACGGAATCGT	60.4	NM_021050	975-995	113 bp
Antisense	CATACCATATCTGTACGGCAGTG	60.3		1087-1065	
Scnn1γ					
Sense	GCACCGACCATTAAGGACCTG	62.7	NM_011326	64-84	118 bp
Antisense	GCGTGAACGCAATCCACAAC	62.8		181-162	
Scnn1β					
Sense	TACCTTGCGGAACTTCACCAG	66.1	NM_011325	603-623	138 bp
Antisense	CAAGCTAGGATTATGCGATCAGG	60.6		740-718	
Scnn1α					
Sense	TACTTCAGCTACCCCGTGAGT	62.6	NM_011324	403-423	153 bp
Antisense	AAAAAGCGTCTGTTCCGTGAT	64.6		555-535	
TNFα					
Sense	ACGTGGAACTGGCAGAAGAG	64.4	NM_013693	182-201	284 bp
Antisense	CTCCTCCACTTGGTGGTTTG	64.5		465-446	
IFNγ					
Sense	GCTCTGACACAATGAACGCT	63.1	NM_008337	99-118	229 bp
Antisense	AAAGAGATAATCTGGCTCTGC	59.1		327-307	
Periostin					
Sense	CACGGCATGGTTATTCCTTCA	60.4	NM_001198766	547-567	151 bp
Antisense	TCAGGACACGGTCAATGACAT	61.1		697-677	
Ctgf					
Sense	CACAGTCATCAACGGAATCGT	60.4	NM_021050	975-995	113 bp
Antisense	CATACCATATCTGTACGGCAGTG	60.3		1087-1065	
mTGFβ1					
Sense	ACTCCACGTGGAAATCAACGG	68.1	NM_011577	693-713	414 bp
Antisense	TAGTAGACGATGGGCAGTGG	62.7	1106-868		
Vegf					
Sense	ATCTTCAAGCCGTCCTGTGTG	66.4	NM_009595	1232-1252	282 bp
Antisense	GCAGGAACATTTACACGTCTG	62.1		1513-1493	
INOS					
Sense	TGGGAATGGAGACTGTCCCAG	66.0	NM_011577	1944-1964	306 bp
Antisense	GGGATCTGAATGTGATGTTTG	60.0		2249-2229	
Mcp-1					
Sense	ATGCAGGTCCCTGTCATGCTTC	69.5	NM_01133	86-107	465 bp
Antisense	ACTAGTTCACTGTCACACTGGTC		68.5	533-511	
αSMA					
Sense	CAGGATGCAGAAGGAGATCAC	64	NM007392.2	1009-1029	364 bp
Antisense	TGTTGCTAGGCCAGGGCTAC	64		1372-1353	
Fn1					
Sense	TTCAAGTGTGATCCCCATGAAG	60	NM_010233	7126-7147	154 bp
Antisense	CAGGTCTACGGCAGTTGTCA	61.5		7279-7260	
Col1α1					
Sense	TAAGGGTCCCCAATGGTGAGA	67.4	NM007742	107-127	203 bp
Antisense	GGGTCCCTCGACTCCTACAT	64.2		309-290	
GAPDH					
Sense	TGGCCTTCCGTGTTCCTAC	61.3	NM_008084	686-704	178 bp
Antisense	GAGTTGCTGTTGAAGTCGCA	60.9		863-844	

Ctfr, cystic fibrosis transmembrane conductance regulator; Scnn1α, sodium channel epithelial 1 α subunit; Scnn1β, sodium channel epithelial 1 β subunit; Scnn1γ, sodium channel epithelial 1γ subunit; TNFα, tumor necrosis factorα; IFNγ, interferonγ; Ctgf, connective tissue growth factor; mTGFβ1, mouse transforming growth factorβ1; Vegf, vascular epithelial growth factor; iNOS, inducible nitric oxide synthase; Mcp-1, monocyte chemoattractant protein-1; αSMA, αsmooth muscle actin; Fn1, fibronectin 1; Col1α1, collagen 1α1. WT, wild-type; TG, transgenic.

Mass spectrometry data with full annotation and e values.

MS/MS Fragmentation of **IVMPESSGNPNAVNPAGYR**
Found in **Staph_8p_00351** in putative transglycosylase IsaA

Match to Query 3631: 1987.945068 from (994.979810,2+) intensity (711086.6200) scans (4233) rawscans (sn4233) rtinseconds (1268.962) index(216)
Title: 217: Scan 4233 (rt=21.1494) [E:\Data\HFX\Isaac Cann\18-154-IsaacCann-Trypsindigested.raw]
Data file E:\Data\HFX\Isaac Cann\18-154-IsaacCann-Trypsindigested.raw

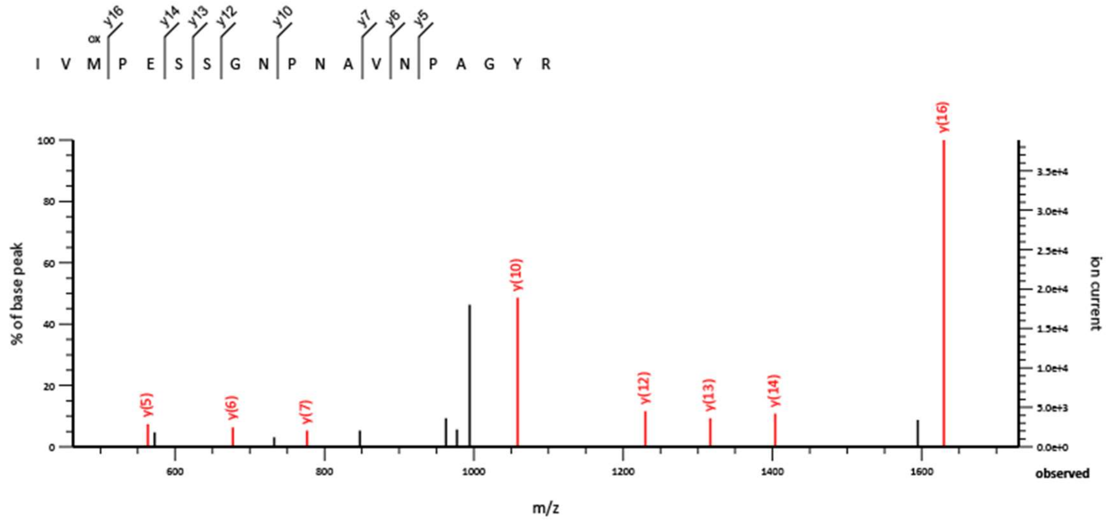

Monoisotopic mass of neutral peptide Mr(calc): 1987.9422
Variable modifications:
M3 : Oxidation (M), with neutral losses 0.0000(shown in table), 63.9983
Ions Score: 80 **Expect:** 1.6e-07
Matches : 9/260 fragment ions using 9 most intense peaks

#	b	b^{++}	b^{*}	b^{*++}	b^{0}	b^{0++}	Seq.	y	y^{++}	y^{*}	y^{*++}	y^{0}	y^{0++}	#
1	114.0913	57.5493					I							19
2	213.1598	107.0835					V	1875.8654	938.4363	1858.8388	929.9231	1857.8548	929.4310	18
3	360.1952	180.6012					M	1776.7970	888.9021	1759.7704	880.3889	1758.7864	879.8968	17
4	457.2479	229.1276					P	***1629.7616***	815.3844	1612.7350	806.8712	1611.7510	806.3791	16
5	586.2905	293.6489			568.2799	284.6436	E	1532.7088	766.8580	1515.6823	758.3448	1514.6982	757.8528	15
6	673.3225	337.1649			655.3120	328.1596	S	***1403.6662***	702.3367	1386.6397	693.8235	1385.6557	693.3315	14
7	760.3546	380.6809			742.3440	371.6756	S	***1316.6342***	658.8207	1299.6076	650.3075	1298.6236	649.8154	13
8	817.3760	409.1917			799.3655	400.1864	G	***1229.6022***	615.3047	1212.5756	606.7914			12
9	931.4190	466.2131	914.3924	457.6998	913.4084	457.2078	N	1172.5807	586.7940	1155.5541	578.2807			11
10	1028.4717	514.7395	1011.4452	506.2262	1010.4612	505.7342	P	***1058.5378***	529.7725	1041.5112	521.2592			10
11	1142.5146	571.7610	1125.4881	563.2477	1124.5041	562.7557	N	961.4850	481.2461	944.4585	472.7329			9
12	1213.5518	607.2795	1196.5252	598.7662	1195.5412	598.2742	A	847.4421	424.2247	830.4155	415.7114			8
13	1312.6202	656.8137	1295.5936	648.3005	1294.6096	647.8084	V	***776.4050***	388.7061	759.3784	380.1928			7
14	1426.6631	713.8352	1409.6366	705.3219	1408.6525	704.8299	N	***677.3365***	339.1719	660.3100	330.6586			6
15	1523.7159	762.3616	1506.6893	753.8483	1505.7053	753.3563	P	***563.2936***	282.1504	546.2671	273.6372			5
16	1594.7530	797.8801	1577.7264	789.3669	1576.7424	788.8748	A	466.2409	233.6241	449.2143	225.1108			4
17	1651.7744	826.3909	1634.7479	817.8776	1633.7639	817.3856	G	395.2037	198.1055	378.1772	189.5922			3
18	1814.8378	907.9225	1797.8112	899.4093	1796.8272	898.9172	Y	338.1823	169.5948	321.1557	161.0815			2
19							R	175.1190	88.0631	158.0924	79.5498			1

Peptide tag	Peptide matches	Protein identification	e-values
1. S.IVMPESSGNPNAVNPAGYR.G + Oxidation (M)	1	Putative transglycosylase IsaA	1.6 e-07
2. T.PNAMANLDVITKKFGASPK.S + Oxidation (M)	1	Sodium/glutamate symporter	0.0025
3. M.FVHLFGLPLP.G	1	Copper-exporting P-type ATPase A	0.014
4. K.LTPPPVK.Q	1	Methionyl-tRNA formyltransferase	0.00031
5. E.PYQSLSELQ.S	1	Spermidine N(1)acetyltransferase	0.0089
6. L.LTIKTYLGG.L	1	Sodium/pantothenate symporter	0.02
7. D.VVIKGHE.R	1	Putative ABC transporter ATP-binding protein YbiT	0.0074
8. N.DISIDSKLKGQV.N	1	Hypothetical protein	0.047
9. F.MFAGKDVLIVYDDLT.K + Oxidation (M)	1	ATP Synthase subunit alpha	0.031
10. L.IAVVLSSAAVSVAGAI.G	3	Iron-uptake system permease protein FeuC	0.044
11. K.PMLVVAFAIIMANTISVI.L + Oxidation (M)	1	Hypothetical protein	0.022
12. S.VPEDAKGQKVFM.E+Oxidation (M)	1	Vitamin B12-binding protein	0.019
13. I.FMMIIGALIGGVTNMIAVRMLFHPFKT.Y + 3 Oxidation (M)	1	Hypothetical protein	0.041
14. A.DKVAKALNKKGSGAGEGSYTYTDMEA.F + Oxidation (M)	1	Macrolide export ATP binding permease MacB	0.037
15. V.SLALPTIRDDLNVTASISLI.F	1	Multidrug efflux pump SdrM	0.04
16. T.EMCKANNVEIAVMI.R + 2 Oxidation (M)	1	CutC-like protein	0.018
17. A.ISGKLPANYADAL.P	1	Transketolase	0.029
18. K.LEKHPYKNPIP.D	1	p-aminobenzyol-glutamate hydrolase subunit B	0.044
19. F.LPKSTEEKHSVARQLNVSVSELEHYIASLN.E	1	Pyruvate, phosphate dikinase	0.027
20. L.LNVTFNFDTNLHTLPPH.F	2	Homoserine dehydrogenase	0.019
21. P.HHEQFVNTTEDIGHQLS.I	1	Putative competence-damage inducible protein	0.029
22. L.YITIDIDGIDPSIAPGTG.T	1	Guanidinobutyrase	0.033
23. E.MTIFEPIKGLIVNK.L + Oxidation (M)	1	Penicillin acylase	0.039
24. M.KVEIGKIINTHG.I	1	Ribosome maturation factor RimM	0.012
25. D.IDGLEVILLVNNNY.K	1	Putative ring cleaving dioxygenase MhqA	0.047
26. Y.VILSDYRGYN.R	1	Hypothetical protein	0.041
27. T.QPVKKGMKEKGVEIVTEAMAKSAE.E + 2 Oxidation (M)	1	Dihyrolipoyl dehydrogenase	0.022
28. G.VLGALEVVEHLNEH.H	1	Putative hydrolase	0.022
29. V.AFILILIHIG.L	1	Hypothetical protein	0.048
30. I.VPVLGPITGGMI.G	1	Glycerol uptake facilitator protein	0.038
31. G.SRPIEQHIKG.F	1	UDP-N-acetylglucosamine 1-carboxyvinyltransferase 1	0.034
32. S.ELTSTLPHAQDYLLR.N	1	Type II secretion system protein F	0.044
33. F.EQSIGFLRIINGSEPLDNTSIH.P	1	30S ribosomal protein S1	0.036
34. H.SDHIKGLGVLARKYGLPI.Y	1	Putative metallo-hydrolase YycJ	0.05
35. R.TGIYMAIDSTNGYMDADRSEWIHD.E + Oxidation (M)	1	C protein alpha-antigen	0.023
36. F.GGYKHSG.I	1	Putative aldehyde dehydrogenase	0.021
37. M.HVTISHP.L	1	Aspartokinase 2	0.031
38. L.LLVSSLLLSQTAMA.A	1	Bifunctional autolysin	0.049
39. K.GILTTIPPRKEIDIVAKVKKAQYNIKKVTQNLY.R	1	Hypothetical protein	0.028
40. V.FIMASILTFA.S + Oxidation (M)	1	Ktr system potassium uptake protein B	0.043
41. N.ieikdep	1	Hypothetical protein	0.025
42. L.EAEEVGNTSFQVFMKT.N + Oxidation (M)	1	Oxygen sensor histidine kinase NreB	0.029
43. S.YMKSMQN.T + Oxidation (M)	1	Putative glycosyltransferase TagX	0.038
44. N. EALKMKKMGVDGG.F+Oxidation (M)	1	Glycerophophodiester phophodiesterase	0.046
45. F.ltINlkfl.Q	1	Amino-acid carrier protein AlsT	0.046

	: icann@illinois.edu
**Search title**	: 18-154-IsaacCann-Trypsindigested.raw-by-no-enzyme-search
**MS data file**	: E:\Data\HFX\Isaac Cann\18-154-IsaacCann-Trypsindigested.raw
	**1:** Cann_largeplas_Staph 20181116 (38 sequences; 6,867 residues)
**Databases**	: **2:** Cann_smallplas_Staph 20181116 (5 sequences; 755 residues)
	**3:** Cann_Staph 20181116 (2,748 sequences; 796,786 residues)
**Timestamp**	: 22 Nov 2018 at 23:27:18 GMT

High performance liquid chromatography data

Chromatographic fractionation of bacterial culture

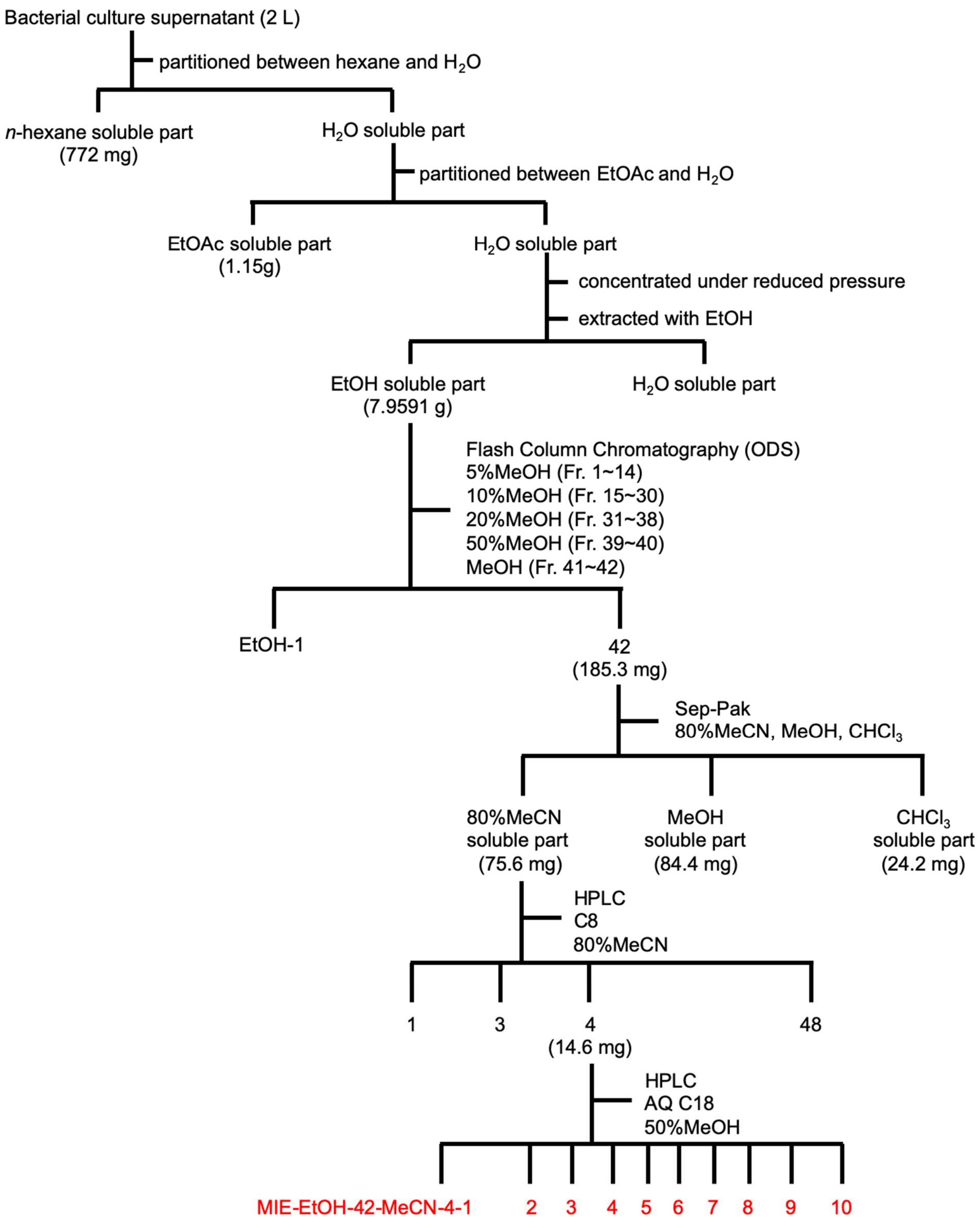

Chromatogram

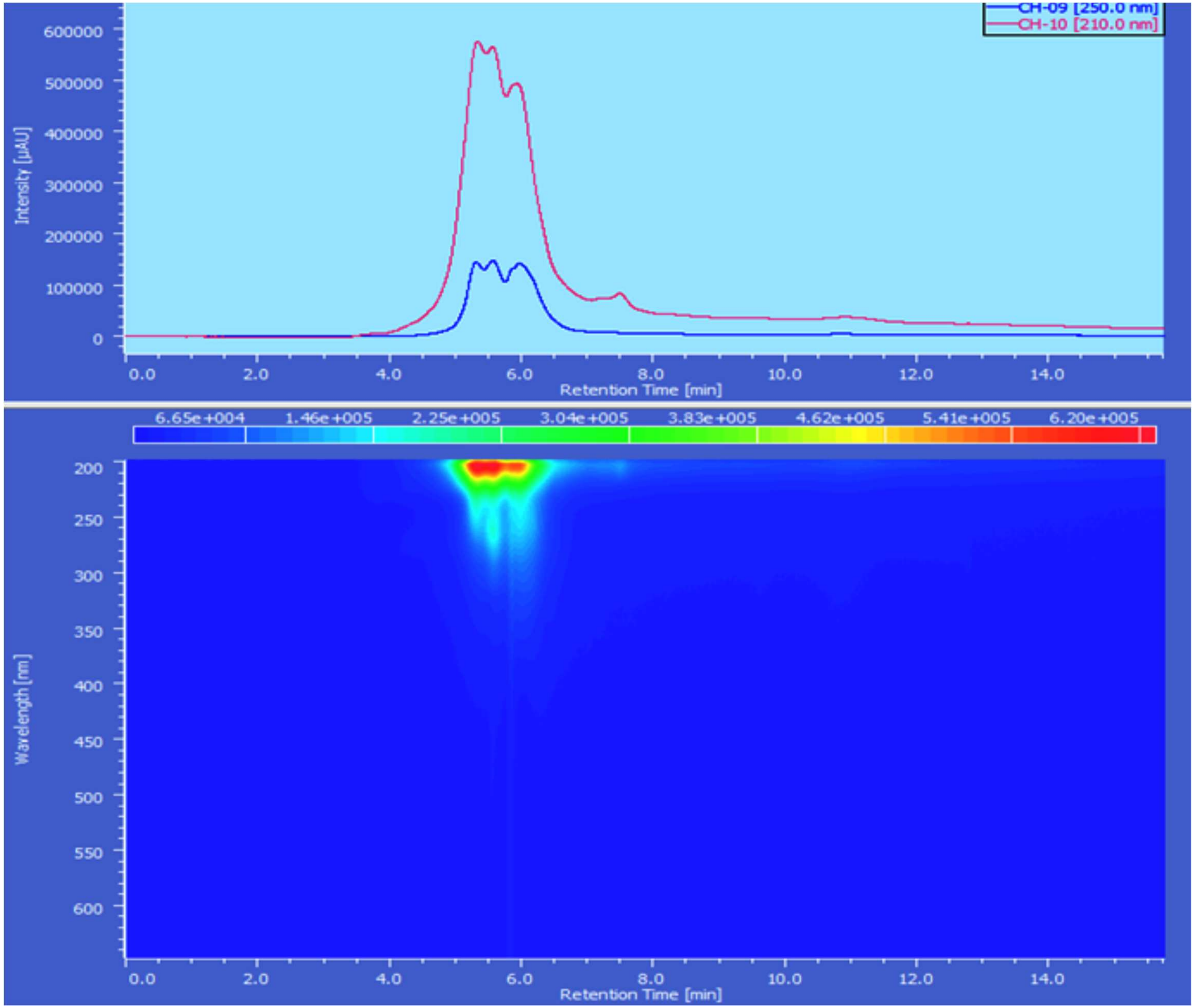

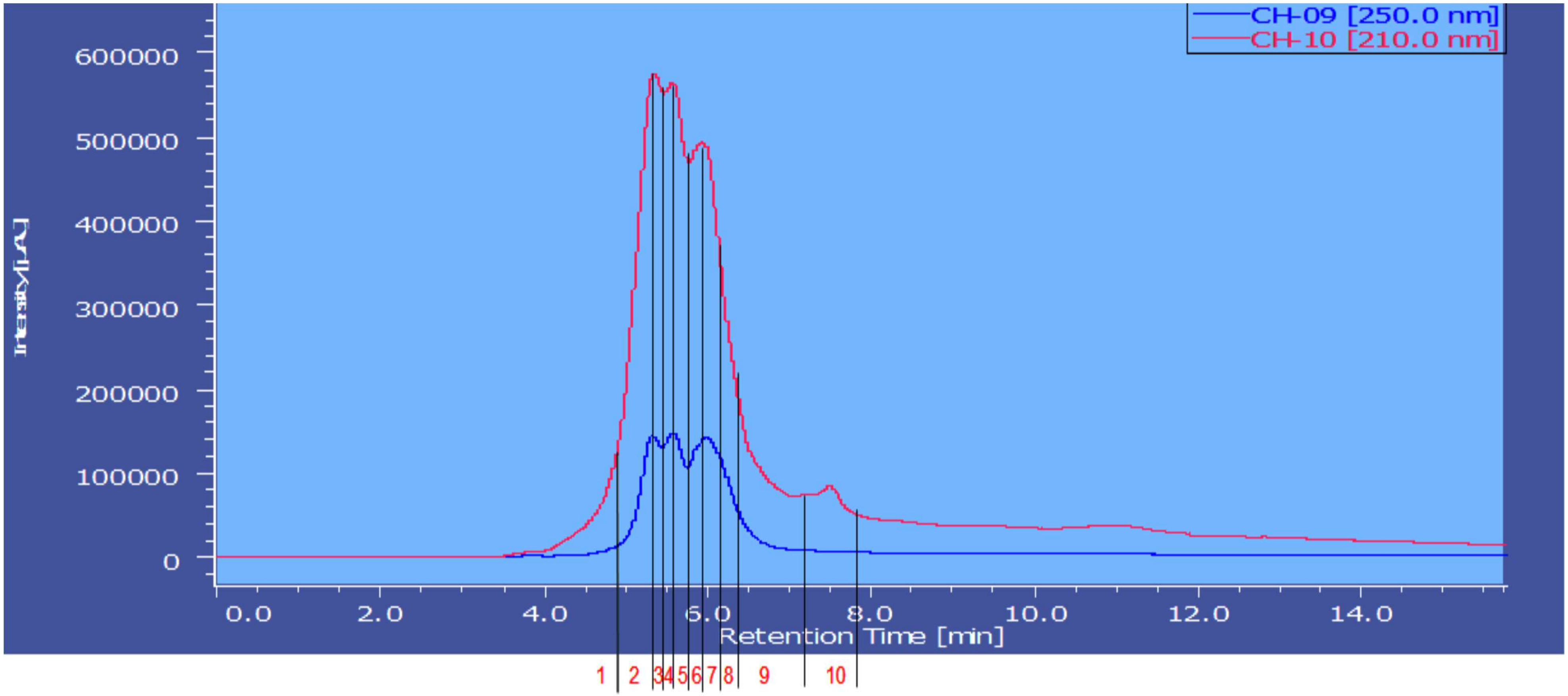

HPLC profiles of MIE-EtOH-42-MeCN-4

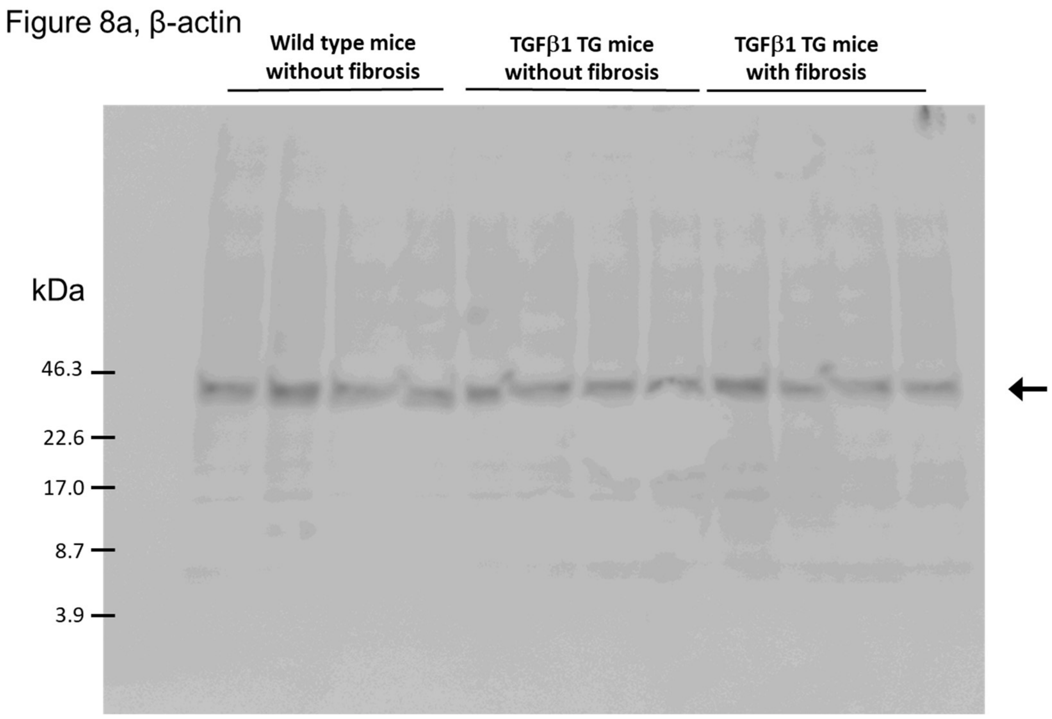

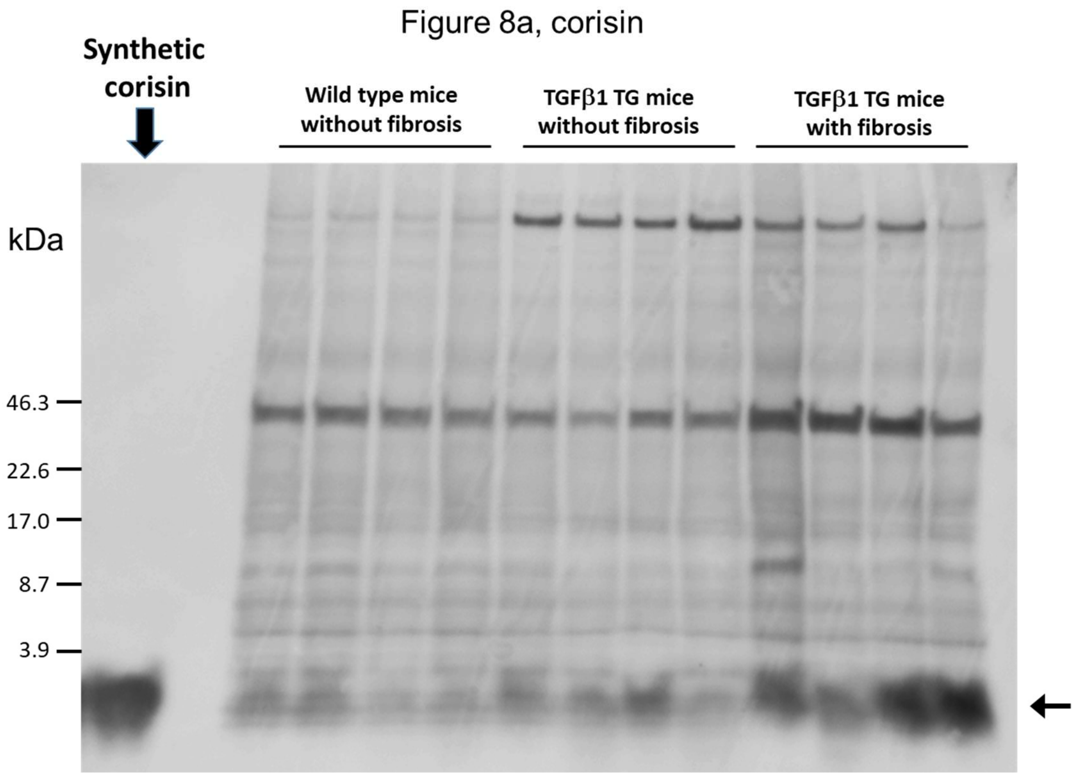

Full blots of portions of blots described in Figure 8a

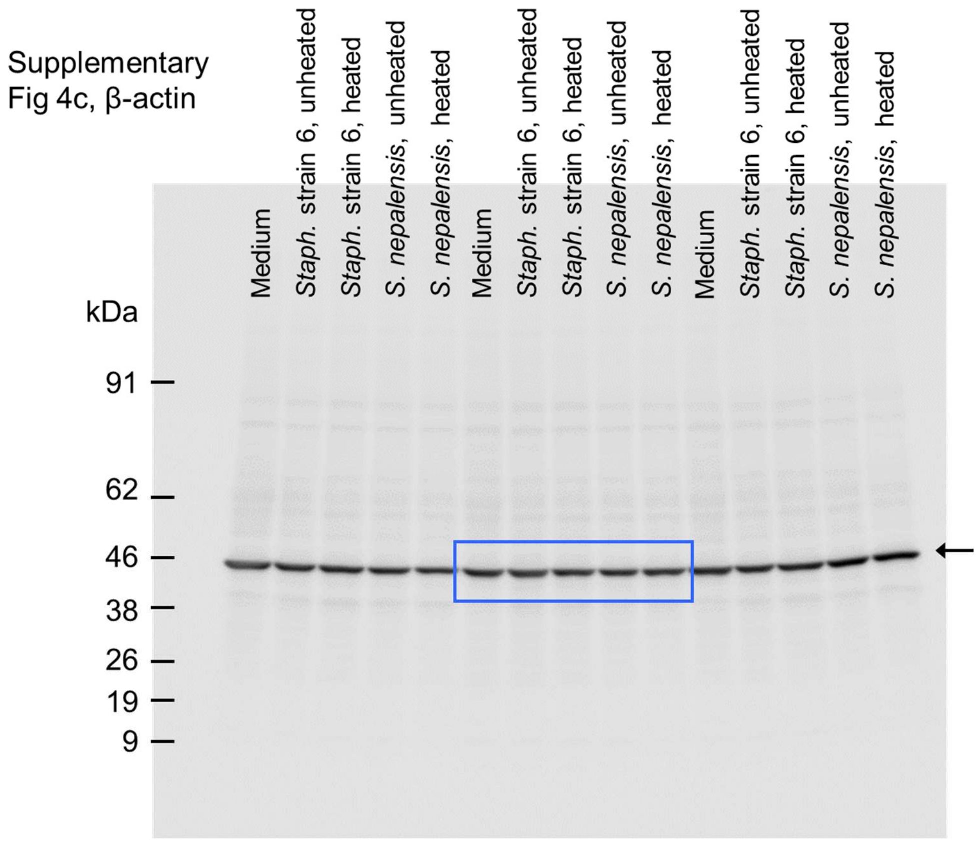

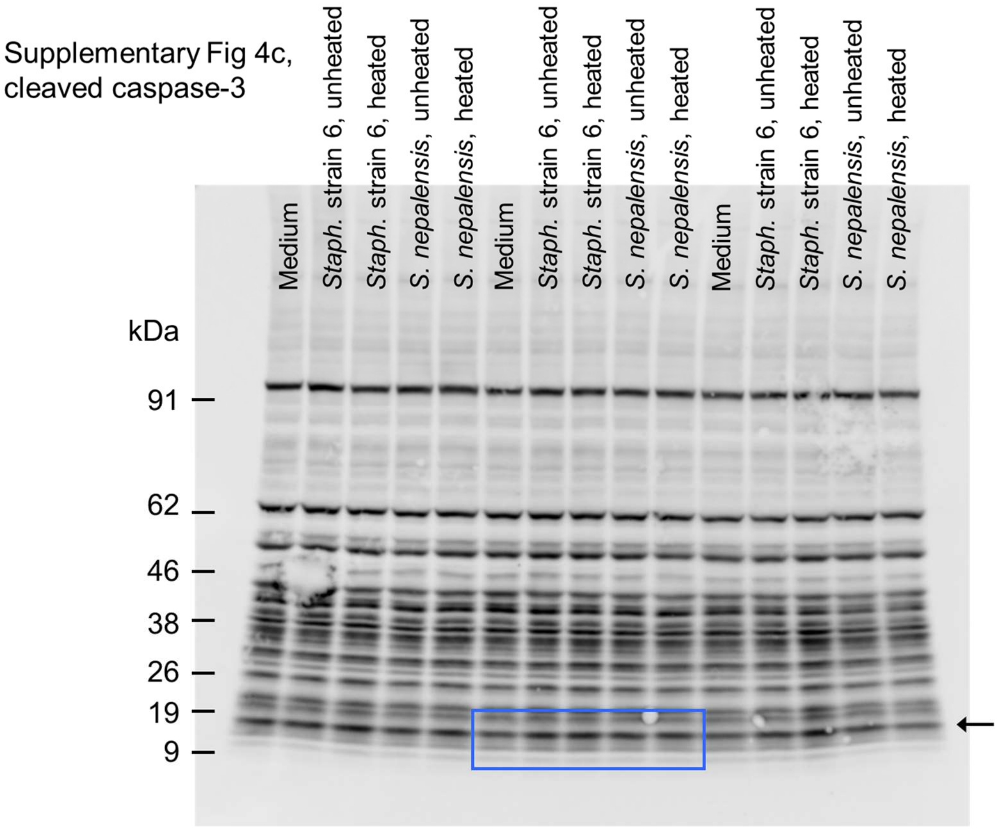

Full blots of portions of blots described in Supplementary Figure 4c

Supplementary Fig 11c, cleaved caspase-3

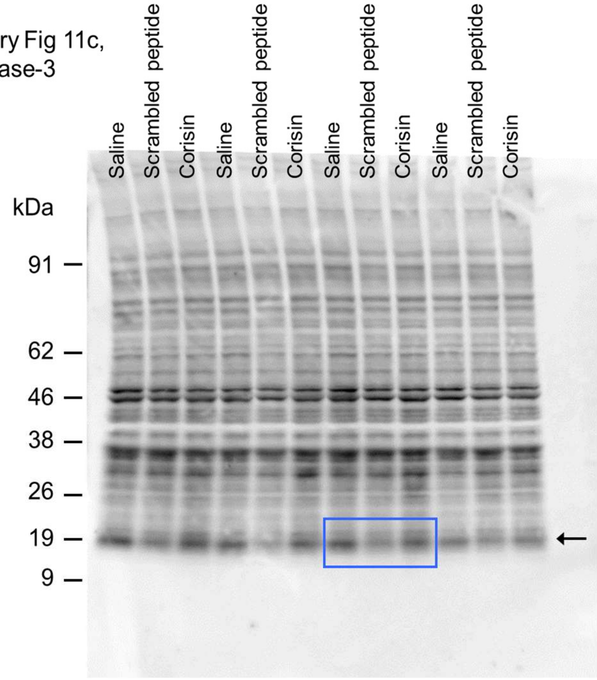

Supplementary Fig 11c, β-actin

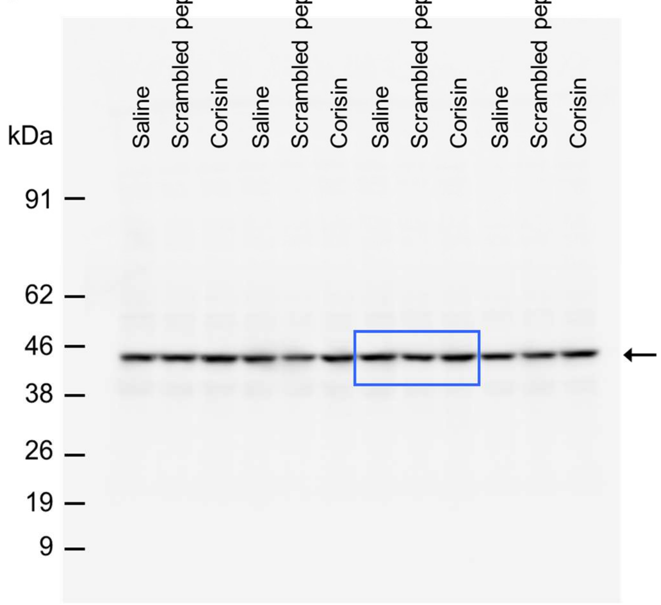

Supplementary Fig 11c, p-Akt

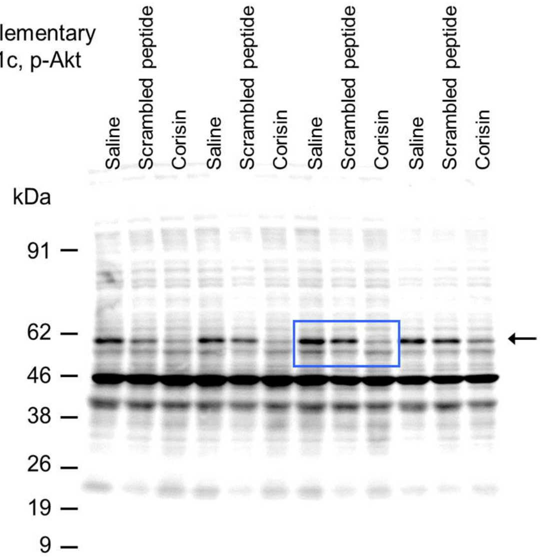

Supplementary Fig 11c, total Akt

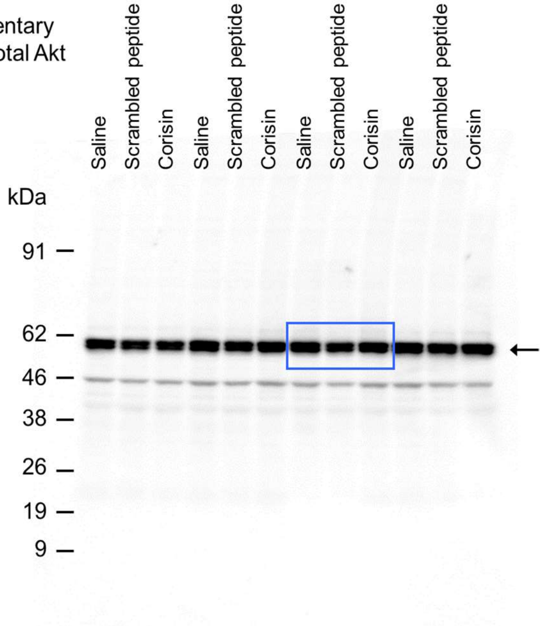

Full blots of portions of blots described in Supplementary Figure 11c

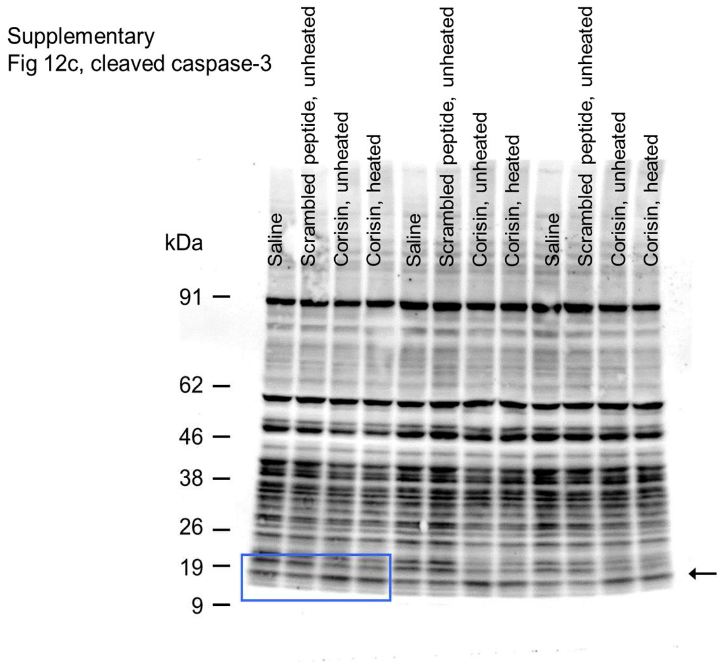

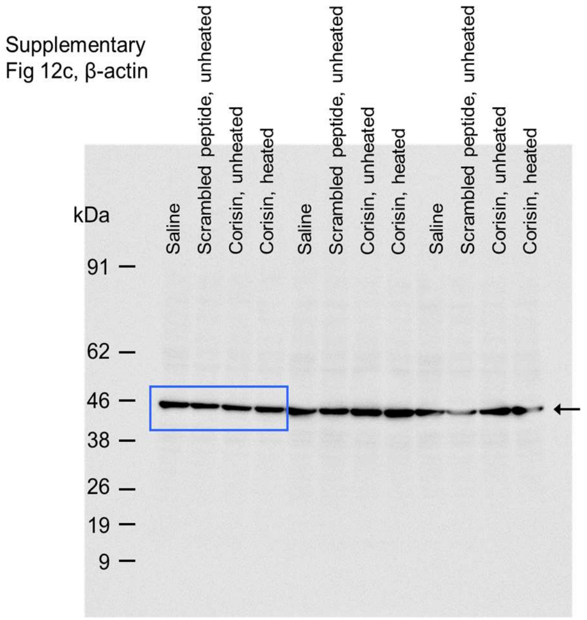

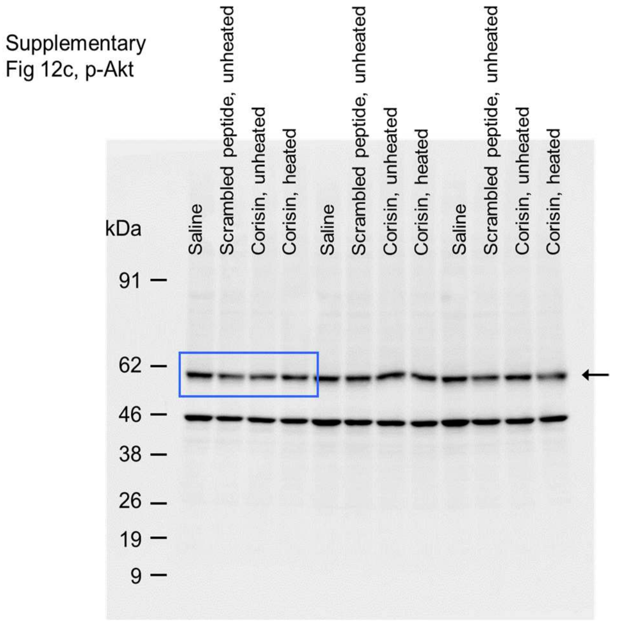

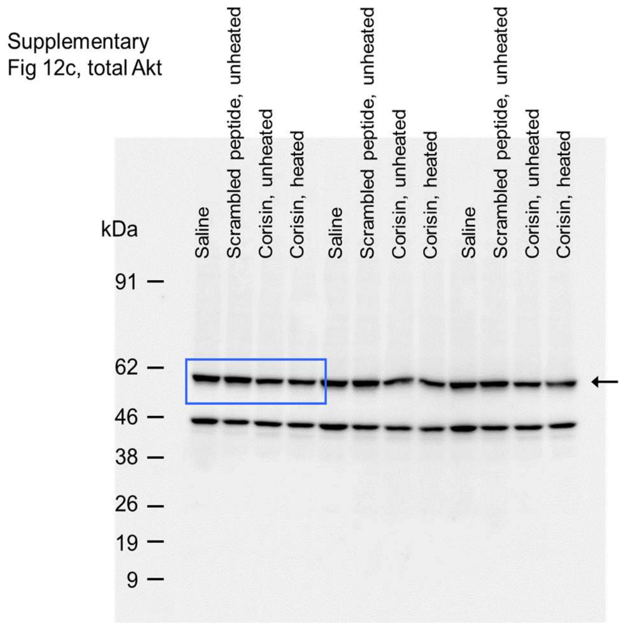

Full blots of portions of blots described in Supplementary Figure 12c
