# Supplementary material for: A Staphylococcus pro-apoptotic peptide induces acute exacerbation of pulmonary fibrosis
Source: Nat Commun. 2020 Mar 24;11:1539. doi: 10.1038/s41467-020-15344-3 (PMC7093394; doi:10.1038/s41467-020-15344-3)
Supplement: Supplementary file 3 — Reporting Summary [file 41467_2020_15344_MOESM3_ESM.pdf]

nature research

Corresponding author(s): Esteban C Gabazza and Isaac Cann

Last updated by author(s): Feb 18, 2020

Reporting Summary

Nature Research wishes to improve the reproducibility of the work that we publish. This form provides structure for consistency and transparency in reporting. For further information on Nature Research policies, see Authors & Referees and the Editorial Policy Checklist.

Statistics

For all statistical analyses, confirm that the following items are present in the figure legend, table legend, main text, or Methods section.

n/a	Confirmed	
☐	☒	The exact sample size (*n*) for each experimental group/condition, given as a discrete number and unit of measurement
☐	☒	A statement on whether measurements were taken from distinct samples or whether the same sample was measured repeatedly
☐	☒	The statistical test(s) used AND whether they are one- or two-sided<br>*Only common tests should be described solely by name; describe more complex techniques in the Methods section.*
☐	☒	A description of all covariates tested
☐	☒	A description of any assumptions or corrections, such as tests of normality and adjustment for multiple comparisons
☐	☒	A full description of the statistical parameters including central tendency (e.g. means) or other basic estimates (e.g. regression coefficient) AND variation (e.g. standard deviation) or associated estimates of uncertainty (e.g. confidence intervals)
☐	☒	For null hypothesis testing, the test statistic (e.g. *F*, *t*, *r*) with confidence intervals, effect sizes, degrees of freedom and *P* value noted<br>*Give P values as exact values whenever suitable.*
☒	☐	For Bayesian analysis, information on the choice of priors and Markov chain Monte Carlo settings
☒	☐	For hierarchical and complex designs, identification of the appropriate level for tests and full reporting of outcomes
☒	☐	Estimates of effect sizes (e.g. Cohen's *d*, Pearson's *r*), indicating how they were calculated

Our web collection on statistics for biologists contains articles on many of the points above.

Software and code

Policy information about availability of computer code

Data collection	The WinROOF software (Mitani Corporation, Tokyo, Japan) for quantification.<br>Olympus BX50 microscope combined with an Olympus DP70 digital camera (Tokyo, Japan) was used for data collection.
Data analysis	The raw mass spectrometry data were analyzed using Mascot 1.6 software.<br>protein sequences were aligned with the MUltiple Sequence Comparison by Log-Expectation (MUSCLE) program .

For manuscripts utilizing custom algorithms or software that are central to the research but not yet described in published literature, software must be made available to editors/reviewers. We strongly encourage code deposition in a community repository (e.g. GitHub). See the Nature Research guidelines for submitting code & software for further information.

Data

Policy information about availability of data

All manuscripts must include a data availability statement. This statement should provide the following information, where applicable:

- Accession codes, unique identifiers, or web links for publicly available datasets
- A list of figures that have associated raw data
- A description of any restrictions on data availability

The datasets generated during and/or analyzed during the current study are available from the corresponding author on reasonable request.

Field-specific reporting

Please select the one below that is the best fit for your research. If you are not sure, read the appropriate sections before making your selection.

☒ Life sciences ☐ Behavioural & social sciences ☐ Ecological, evolutionary & environmental sciences

For a reference copy of the document with all sections, see nature.com/documents/nr-reporting-summary-flat.pdf

Life sciences study design

All studies must disclose on these points even when the disclosure is negative.

Sample size	The number of samples for each set of experiment was determined based on previous experience and standards in the field.
Data exclusions	No exclusion of data was done.
Replication	All measurements were reproducible and all attempts of replications were successful.
Randomization	Mice were randomized for group allocation.
Blinding	Some sets of data (CT finding scoring, lung fibrosis scoring, collagen deposition) have been analyzed in a double-blind approach. The authors measuring parameters in samples were unaware of the treatment.

Reporting for specific materials, systems and methods

We require information from authors about some types of materials, experimental systems and methods used in many studies. Here, indicate whether each material, system or method listed is relevant to your study. If you are not sure if a list item applies to your research, read the appropriate section before selecting a response.

Materials & experimental systems

n/a	Involved in the study	
☐	☒	Antibodies
☐	☒	Eukaryotic cell lines
☒	☐	Palaeontology
☐	☒	Animals and other organisms
☐	☒	Human research participants
☐	☒	Clinical data

Methods

n/a	Involved in the study	
☐	☒	ChIP-seq
☐	☒	Flow cytometry
☒	☐	MRI-based neuroimaging

Antibodies

Antibodies used	Mouse Ly-6G/Ly-6C, FITC, clone RB6-8C5, rat, IgG2bk<br>Mouse F4/80, CIA3-1, rat, IgG2bk<br>Mouse CD11c, PE/Cy5, clone N418, hamster, IgG<br>Mouse CD3[illegible], FITC, 145-2C11, hamster, IgG<br>Mouse CD45R/B220, PE/Cy5, RA3-6B2, rat, IgG2ak<br>Anti-mouse CD25, FITC, PC61, rat, IgG1k<br>Mouse CD8a, PE, 53-6.7, rat, IgG2ak<br>mouse CD4, PE/Cy5, GK1.5, rat, IgG2bk<br>mouse NK1.1, PE, PK136, mouse, IgG2bk<br>Annexin V, FITC
Validation	Antibodies were validated by the manufacturers (BioLegend Inc or BD Pharmingen).

Eukaryotic cell lines

Policy information about cell lines

Cell line source(s)	Cell lines were from the American Type Culture Collection (Manassas, VA).
Authentication	Expression of specific markers were evaluated for authentication.
Mycoplasma contamination	There was no Mycoplasma contamination.
Commonly misidentified lines (See ICLAC register)	Commonly misidentifed cell line was not used.

Animals and other organisms

Policy information about studies involving animals; ARRIVE guidelines recommended for reporting animal research

Laboratory animals	Mice (males and females) aging between 12 and 20 weeks with C57Bl6/j background were used.
Wild animals	Wild type (non-transgenic) mice were provided by Japan SCL (Hamamatsu, Japan).
Field-collected samples	All mice were maintained in a specific pathogen-free environment under a 12-h light/dark cycle in an institutional facility for experimental animals.
Ethics oversight	The Recombinant DNA Experiment Safety Committee and the Committee for Animal Investigation of Mie University approved the experimental protocols and all procedures were performed in accordance with internationally approved principles of laboratory animal care published by the National Institute of Health (https://olaw.nih.gov/).

Note that full information on the approval of the study protocol must also be provided in the manuscript.

Human research participants

Policy information about studies involving human research participants

Population characteristics	This study comprised 34 patients with stable idiopathic pulmonary fibrosis and eight male healthy volunteers.
Recruitment	Patients and healthy subjects that gave written informed consent were included in the study.
Ethics oversight	The study protocol was approved by the Ethical Committees for Clinical Investigation of Mie University, Matsusaka Municipal Hospital, and Chuo Medical Center and conducted following the Principles of the Declaration of Helsinki.

Note that full information on the approval of the study protocol must also be provided in the manuscript.

Clinical data

Policy information about clinical studies

All manuscripts should comply with the ICMJE guidelines for publication of clinical research and a completed CONSORT checklist must be included with all submissions.

Clinical trial registration	This was not a clinical trial.
Study protocol	This was not a clinical trial.
Data collection	Blood samples were taken after informed consent was obtained from the subjects.
Outcomes	The persons that measured parameters in the subjects were unaware of the groups of subjects.

ChIP-seq

Data deposition

☒ Confirm that both raw and final processed data have been deposited in a public database such as GEO.

☒ Confirm that you have deposited or provided access to graph files (e.g. BED files) for the called peaks.

Data access links<br>*May remain private before publication.*	https://www.ncbi.nlm.nih.gov/nuccore/?term=PRJNA544423
Files in database submission	The whole DNA sequence of cultured bacteria
Genome browser session<br>(e.g. UCSC)	not applicable

Methodology

Replicates	All replicates were reproducible.
Sequencing depth	The majority of the reads were 6 kb to 30 kb, although reads as long as 94 kb were also obtained.
Antibodies	*Describe the antibodies used for the ChIP-seq experiments; as applicable, provide supplier name, catalog number, clone name, and lot number.*
Peak calling parameters	Genomic DNA from the bacterial strain (400 ng) was converted into a Nanopore library with the Rapid Barcoding library kit

Peak calling parameters	SQK-RAD004. The library was sequenced on a SpotON R9.4.1 FLO-MIN106 flowcell for 48h on a GridION sequencer. Base-calling was performed with Guppy 1.4.3, and demultiplexing was done with Porechops 0.2.3.
Data quality	A workflow was developed to perform four assemblies as follows, primarily to assess quality using different assembly strategies to find the best overall assembly. Initial assembly of the Oxford Nanopore data was carried out using Canu, followed by polishing using Nanopolish and Pilon (utilizing the Illumina MiSeq reads), and finally the genome was re-oriented using Circlator.
Software	The Illumina Miseq sequencing was carried out by preparing shotgun genomic libraries with the Hyper Library construction kit from Kapa Biosystems (Roche). The library was quantitated by qPCR and sequenced on one MiSeq Nano flowcell for 251 cycles from each end of the fragments using a MiSeq 500-cycle sequencing kit version 2. Fastq files were generated and demultiplexed with the bcl2fastq v2.20 Conversion Software (Illumina).

Flow Cytometry

Plots

Confirm that:

☒ The axis labels state the marker and fluorochrome used (e.g. CD4-FITC).

☒ The axis scales are clearly visible. Include numbers along axes only for bottom left plot of group (a 'group' is an analysis of identical markers).

☒ All plots are contour plots with outliers or pseudocolor plots.

☒ A numerical value for number of cells or percentage (with statistics) is provided.

Methodology

Sample preparation	A549 and normal human bronchial epithelial cells were cultured in DMEM supplemented with 10% fetal calf serum and antibiotics at 5% CO_2 atmosphere at 37 °C. The cells were washed and then treated with propidium iodide and annexin V to evaluate apoptosis or with the specific antibody to evaluate specific cell population.
Instrument	A flow cytometer (FACScan) from BD Biosciences was used.
Software	Cellquest software from BD Biosciences was used for data analysis.
Cell population abundance	Sufficient number of cells was used in each experiment (500,000 to 1,500,000 cells per experiment).
Gating strategy	Gates were placed around cell populations with common characteristics after determining forward scatter, side scatter and marker expression.

☒ Tick this box to confirm that a figure exemplifying the gating strategy is provided in the Supplementary Information.
